# Accurate control of dual-receptor-engineered T cell activity through a bifunctional anti-angiogenic peptide

**DOI:** 10.1186/s13045-018-0591-7

**Published:** 2018-03-20

**Authors:** Erhao Zhang, Jieyi Gu, Jianpeng Xue, Chenyu Lin, Chen Liu, Mengwei Li, Jingchao Hao, Sarra Setrerrahmane, Xiaowei Chi, Weiyan Qi, Jialiang Hu, Hanmei Xu

**Affiliations:** 1 The Engineering Research Center of Peptide Drug Discovery and Development, China Pharmaceutical University, Nanjing, 210009 People’s Republic of China; 2 State Key Laboratory of Natural Medicines, Ministry of Education, China Pharmaceutical University, Nanjing, 210009 People’s Republic of China; 3 School of Pharmaceutical Sciences and Yunnan Provincial Key Laboratory of Pharmacology for Natural Products, Kunming Medical University, Kunming, 650500 People’s Republic of China; 4 Nanjing Anji Biotechnology Co., Ltd., Nanjing, 210046 People’s Republic of China

**Keywords:** Cancer immunotherapy, Switchable dual-receptor CAR, K562 cell, HM-3, Bifunctional molecule

## Abstract

**Background:**

Chimeric antigen receptors (CARs) presented on T cell surfaces enable redirection of T cell specificity, which has enormous promise in antitumor therapy. However, excessive activity and poor control over such engineered T cells cause significant safety challenges, such as cytokine release syndrome and organ toxicities. To enhance the specificity and controllable activity of CAR-T cells, we report a novel switchable dual-receptor CAR-engineered T (sdCAR-T) cell and a new switch molecule of FITC-HM-3 bifunctional molecule (FHBM) in this study.

**Methods:**

We designed a fusion molecule comprising FITC and HM-3. HM-3, an antitumor peptide including an Arg-Gly-Asp sequence, can specifically target integrin αvβ3 that is presented on some tumor cells. Moreover, to improve the specificity of CAR-T cells, we also generated the sdCAR-T cell line against cognate tumor cells expressing human mesothelin (MSLN) and integrin αvβ3. Finally, the activity of sdCAR-T cell and FHBM is verified via in vitro and in vivo experiments.

**Results:**

In the presence of FHBM, the designed sdCAR-T cells exerted high activity including activation and proliferation and had specific cytotoxicity in a time- and dose-dependent manner in vitro. Furthermore, using a combination of FHBM in nude mice, sdCAR-T cells significantly inhibited the growth of MSLN^+^ K562 cells and released lower levels of the cytokines (e.g., interleukin-2, interferon γ, interleukin-6, and tumor necrosis factor α) relative to conventional CAR-T cells, obtaining specific, controllable, and enhanced cytotoxicity.

**Conclusions:**

Our data indicate that FHBM can accurately control timing and dose of injected CAR-T cells, and sdCAR-T cells exert significant antitumor activity while releasing lower levels of cytokines for the cognate tumor cells expressing both MSLN and integrin αvβ3. Therefore, combination therapies using sdCAR-T cells and the switch molecule FHBM have significant potential to treat malignancies.

**Electronic supplementary material:**

The online version of this article (10.1186/s13045-018-0591-7) contains supplementary material, which is available to authorized users.

## Background

The adoptive transfer of genetically engineered T cells expressing a chimeric antigen receptor (CAR) specific for the tumor antigen CD19 has been highly successful in the treatment of some human malignancies, including leukemia, lymphoma, and other hematological cancers [1–6]. CAR-engineered T cells, a novel form of cancer immunotherapy, marked the beginning of a new era in the war between the immune system and the tumor and have provided an effective platform to combat cancers [7–10]. However, the application of current CAR-T cell therapy is associated with some serious side toxicities, such as cytokine release syndrome (CRS) and organ toxicities, resulting from excessive activity and poor control [11–13]. To address these safety concerns while retaining potent cytotoxicity against cognate tumor cells, several innovative strategies recently have been described that regulate the selectivity and activity of CAR-T cells, including the suicide gene [14, 15], inhibitory CAR [16], dual-antigen receptor [17], and an exogenous molecule used as a switch to control CAR-T cell activity [18–21].

To improve the controllable activity of injected CAR-T cells, one approach is to generate a dual-receptor mode for engineered T cells that exerts cytotoxicity against tumor cells that simultaneously express two distinct antigens. Kloss et al. created dual-receptor-engineered T cells that have specific activity against the prostate tumors expressing both prostate stem cell antigen and prostate-specific membrane antigen [17]. Another approach is to engineer a switch molecule to control the activity of CAR-T cells. For instance, Wu et al. recently proposed a novel design, termed an ON-switch CAR, that can eliminate cognate target cells in the presence of an exogenous molecule, AP21967 [18]. Kim et al. provided another switch molecule consisting of folate and fluorescein isothiocyanate (folate-FITC) that can regulate the cytotoxicity of FITC-specific CAR-T cells toward tumor cells expressing folate receptors [21]. Although the ability of CAR-T cells to recognize tumor cells and normal cells has improved based on dual-receptor CARs, their excessive activity is not effectively controlled. In contrast, switch molecules can effectively control the dose of activated CAR-T cells. To date, there is no treatment paradigm of dual-receptor-modified T cells in combination with switch molecules in the field of cancer immunotherapy. Therefore, we propose a new CAR method using a dual-receptor model to enhance the efficacy and safety of T cells by introducing a novel bifunctional molecule. Compared with using a single molecule as a switch, a bifunctional molecule significantly improves the therapeutic effects of CAR-T cells by constitutive antitumor activity.

To generate engineered T cells with specific cytotoxicity for tumor cells, a wide variety of potential tumor targets are under consideration, such as mesothelin (MSLN), carcinoembryonic antigen (CEA), epidermal growth factor receptor, human epidermal growth factor receptor 2, and so on [22]. In particular, the tumor antigen MSLN is emerging as an attractive target for cancer immunotherapy, considering its high expression on various tumors, including mesothelioma, ovarian, lung, esophageal, pancreatic, gastric, and breast cancers, which facilitates MSLN-specific CAR-T cells to eliminate these cancers [23, 24]. In addition, our previous studies have shown that the HM-3 molecule is a novel antitumor peptide composed of 18 amino acids, including an Arg-Gly-Asp sequence, which specifically targets integrin αvβ3-expressing tumor cells [25]. In vivo and in vitro studies have shown that HM-3 suppresses tumor growth by anti-angiogenesis [26]. Therefore, a bifunctional molecule consisting of FITC coupled with HM-3 can serve as a novel switch to precisely regulate the specificity and controllable cytotoxicity of CAR-T cells.

Here, we designed a fusion molecule of HM-3 and FITC, termed FITC-HM-3 bifunctional molecule (FHBM), as a switch to accurately control timing and dose of injected CAR-T cells. Moreover, to improve the specificity of CAR-T cells, we also generated a switchable dual-receptor CAR-T (sdCAR-T) cell line that targets both the antigen MSLN and integrin αvβ3 via the switch molecule FHBM, which we described in our previously published review [27]. Our results demonstrated that sdCAR-T cells exert significant antitumor activity while releasing lower levels of cytokines in the presence of both cognate tumor cells and FHBM that by itself has an inhibitory effect on tumor angiogenesis, thereby improving the application of CAR-T cell therapy. Therefore, the combination therapy using sdCAR-T cells and bifunctional molecules is becoming increasingly prospective in cell-based therapies for malignancies and other diseases.

## Methods

### Isolation, identification, and culturing conditions of human T cells

All samples were collected under a protocol approved by the Ethics Committee of China Pharmaceutical University, following written informed consent. Peripheral blood mononuclear cells (PBMCs) were isolated from an anonymous healthy donor’s blood by Lymphoprep™ reagent (Axis-Shield). Isolated CD4^+^ T or CD8^+^ T cells were immediately enriched by positive selection with magnetic microbeads (Miltenyi Biotec). The two isolated T cell subtypes were cultured in human T cell complete medium, consisting of X-VIVO15 (Lonza), 10% (*v*/*v*) fetal bovine serum (FBS, Biological Industries), and 10 mM N-acetyl L-Cysteine (Sigma-Aldrich), supplemented with 40 and 80 IU/mL of recombinant human IL-2 (PeproTech) for CD4^+^ T and CD8^+^ T cells, respectively. The sorted T cells were resuspended in phosphate buffer saline (PBS) and stained with an anti-CD4-PE (phycoerythrin) antibody (BD Pharmingen) or an anti-CD8-APC (allophycocyanin) antibody (BD Pharmingen). The control groups were designed and stained with an isotype antibody IgG1κ-PE (BD Pharmingen) or IgG1κ-APC (BD Pharmingen), respectively. All stained cells were washed three times in PBS and processed with a flow cytometer.

### Engineering of T cells by electroporation and verification of sdCAR expression

The φC31 integrase, a member of the serine integrase system, is a sequence-specific recombinase that mediates recombination between an *att*B site containing a donor plasmid and a pseudo-*att*P site within the target genome. In this research, sdCAR gene with an *att*B site was unidirectionally integrated into a T cell genome by a φC31 integrase at sites with a pseudo-*att*P sequence. The isolated CD8^+^ T cells or CD4^+^ T cells were mixed with some plasmids including sdCAR vectors and φC31 integrase vectors at a 1:50 ratio (*m*/*m*). Finally, T cells were electroporated under the electroporation condition of F1-115 program according to the manufacturer’s instructions. Then, the engineered CAR-T cells had been tested by sequencing for correctness (GenScript (Nanjing) Co., Ltd.). Expression of sdCAR structure on the surface of effector cells was verified by detection of BFP expression with a flow cytometer. In addition, BFP expression was also detected by western blot with the use of an anti-BFP antibody (BioVision).

### Lentiviral engineering of target cells and detection with flow cytometry

A gene sequence containing the human MSLN (accession number NM_006665) and GFP (accession number GU452685) was synthesized by GENEWIZ® company and cloned into the pLV-vector (Hanbio Biotechnology Co., Ltd.). At the same time, a non-cognate tumor antigen gene, containing the sequence for CEA (accession number M29540), and another fluorescent protein gene mCherry (accession number KT894026) was also constructed. The engineered K562 cells after lentiviral transduction were cultured in RPMI-1640 medium supplemented with 10% FBS, 100 U/mL penicillin, and 100 μg/mL streptomycin (Sangong Biotech). In order to maintain the expression of the tumor antigen, engineered-K562 cells were cultured in medium containing 0.25 μg/mL puromycin (Sangong Biotech) or 50 μg/mL hygromycin B (Sangong Biotech) for selection of MSLN- or CEA-expressing K562 cells, respectively. In addition, the engineered HT29 cells expressed the cognate tumor antigen (MSLN) by lentiviral transduction and then selectively cultured in complete medium supplemented with 0.5 μg/mL puromycin. AsPC-1 cells were genetically engineered to express the red fluorescent protein (RFP) by lentiviral transduction. Expression of tumor antigens was detected with Western blot in which target proteins were probed with an anti-MSLN antibody (R&D Systems) or an anti-CEA antibody (R&D Systems).

### Generation of the novel switch molecule FHBM

The switch molecule FHBM was generated using FluoroTag™ FITC Conjugation Kit (Sigma, #FITC1). Briefly, solute FITC and HM-3 peptide were mixed at the molar ratio of 5:1 and incubated for 2 h at room temperature in a reaction vial with gentle stirring and covering with aluminum foil to protect from light. Then, the conjugated product was purified from the unconjugated FITC molecule by size filtration with a separation column prepacked with Sephadex G-25M (Sephadex G-25M column) in order to obtain a higher purity of FHBM. Finally, FHBM was analyzed by HPLC with an isocratic mobile phase consisting of 50% acetonitrile and 50% water containing 0.1% trifluoroacetic acid. The injection volume was 10 μL, the flow rate at the mobile phase was 1.0 mL/min, and chromatography was performed at 25 °C. The molecular weight of FHBM was confirmed by mass spectrometry. In addition, the FITC/HM-3 molar ratio and the concentration of the final conjugate were determined using a spectrophotometer, calculating with some formulas involved in the manufacturer’s instruction.

### sdCAR-T cell activation assays in vitro

In a U-bottom 96-well plate, the CD4^+^ T cells expressing the sdCAR were incubated with cognate or non-cognate antigen-expressing target cells at an effector:target cell ratio of 1:2 in varying types of switch molecules including PBS, HM-3, and FHBM, respectively. Simultaneously, a positive control was designed, including a second generation of CAR specific for MSLN (MζBB CAR), to assess the sdCAR-T cell cytotoxicity. After incubation for 24 h, the supernatant was harvested, and the levels of interleukin-2 (IL-2) or interferon γ (IFNγ) were measured using the human IL-2 or IFNγ ELISA kit (MultiSciences), respectively. At the same time, the cells were resuspended in PBS and stained with an APC-conjugated anti-human CD69 antibody (BioLegend). Stained cells were washed three times with PBS and finally resuspended in 200 μL PBS. CD69 expression was detected with a flow cytometry.

### sdCAR-T cell proliferation assay in vitro

Proliferation activity of sdCAR-engineered CD4^+^ T cells was assessed by counting of total number of cells and the percentage of sdCAR-engineered T cells using a cell counter and flow cytometry, respectively. K562 target cells expressing the cognate antigen (MSLN) did not proliferate under the treatment with 25 μg/mL mitomycin C (MedChem Express) for 30 min at 37 °C. In a U-bottom 96-well plate, sdCAR-T cells (or MζBB CAR-T cells) and target cells were mixed at 1:2 ratio in the presence of 100 pM exogenous molecules. At the same time, the cells in the vehicle group were incubated in PBS without exogenous molecules. For blue fluorescent protein (BFP) detection, the total cells were collected for flow cytometry and cell counter analysis after incubation for 3, 4, and 5 days.

### sdCAR-T cell cytotoxicity assay in vitro

For the specific cytotoxicity in vitro, the cytolytic activity was measured by the amount of LDH (lactate dehydrogenase) released into cultured media using LDH Cytotoxicity Assay Kit (Cayman chemical). For other experiments, unless otherwise stated, the cytolytic activity of sdCAR-engineered CD8^+^ T cells was assessed using a flow cytometry-based cytotoxicity assay [18]. Attributed to the differential expression of green fluorescent protein (GFP) and mCherry on cognate and non-cognate K562 cells, the two types of cells could be simultaneously detected by flow cytometry in samples of each treatment condition. In this experiment, the growth state of CEA^+^ K562 cells is similar to MSLN^+^ K562 cells without the sdCAR-T cell cytotoxicity. In the in vitro assays, a cell mixture containing two tumor cell lines at a 1:1 ratio were co-incubated with sdCAR-CD8^+^ T cells at a T cell:target cell ratio of 5:1 in a U-bottom 96-well plate. The exogenous molecules, HM-3 or FHBM, were added at a final concentration of 100 pM in each reaction well. After a 22-h incubation and centrifugation at 800×*g* for 5 min, the pelleted cells were washed three times with PBS and finally resuspended in 200 μL PBS for flow cytometry analysis. For each reaction sample, the survival rate of cognate target cell was represented as a ratio of the surviving MSLN^+^ K562 cells to CEA^+^ K562 cells. The cytotoxic activity of sdCAR-T cells was enumerated based on the cognate target cell survival.

### In vivo cytotoxic effect of sdCAR-T cells

Female NOD.CB17-Prkdc^scid^/NcrCrl (NOD SCID) mice, 6–8 weeks of age, were purchased from Charles River Laboratories (Beijing Vital River Laboratory Animal Technology Co., Ltd.) and cared by the veterinary staff. All procedures were performed as approved by the Institutional Animal Care and Use Committee of China Pharmaceutical University. A target cell mixture of MSLN^+^ K562 cells and CEA^+^ K562 cells (1 × 10^7^ cells per cell type) was resuspended in 2 mL PBS and then injected into the intraperitoneal (i.p.) space of each nude mouse. All mice were randomly divided into six groups (five mice per group): untransduced T cell (No CAR), MζBB CAR-T cell, and sdCAR-T cells with three kinds of molecule switches (vehicle/PBS, HM-3, or FHBM). Twelve hours later, the effector cells (~ 1 × 10^7^ cells) including T cells, conventional CAR-T cells, and sdCAR-T cells were injected i.p., followed by injection of FHBM (at 0.5 mg/kg dosage), HM-3 (at 0.5 mg/kg dosage), or PBS (vehicle group, ~ 110 μL). Thirty-six hours after the exogenous molecule or PBS injection, the mice were euthanized using a two-step euthanasia method including carbon dioxide asphyxiation followed by cervical dislocation. The tumor cells were re-suspended in 5 mL cold PBS (with 3% FBS, *v*/*v*) in the peritoneal cavity of mice. The peritoneal lavage fluid was collected as much as possible and kept on ice. Briefly, the obtained peritoneal lavage was centrifuged at 800×*g* for 8 min, and the collected cells were re-suspended in 1 mL red blood cell lysis solution for 30 min at room temperature to avoid the blood pollution. The supernatant derived from the peritoneal fluid was analyzed for cytokine release, such as IL-2, IFNγ, interleukin-6 (IL-6), and tumor necrosis factor α (TNFα). After centrifugation again at 800×*g* for 8 min, the collected cells were resuspended in 200 μL PBS for flow cytometry analysis according to the methods described in cytotoxicity assays in vitro.

For the solid tumor models, the nude mice were injected with engineered AsPC-1 cells (5 × 10^6^ cells) intravenously. On day 7, 1 × 10^7^ sdCAR-T cells (or MζBB CAR-T cells) were infused and switches were dosed 6 h later intravenously. On day 10 and day 20 after engineered T cell injection, the tumor burden was measured by IVIS.

### Statistical analysis

For single comparisons, a two-tailed Student’s *t* test was used. The *n* values used to calculate statistics are indicated in figure legends. All experiments were replicated at least three times. The symbol n.s. indicates not significant and the symbols *, **, and *** indicate *P* values less than 0.05, 0.01, and 0.001, respectively.

## Results

### Design features of the sdCAR structure

**Fig. 1:**
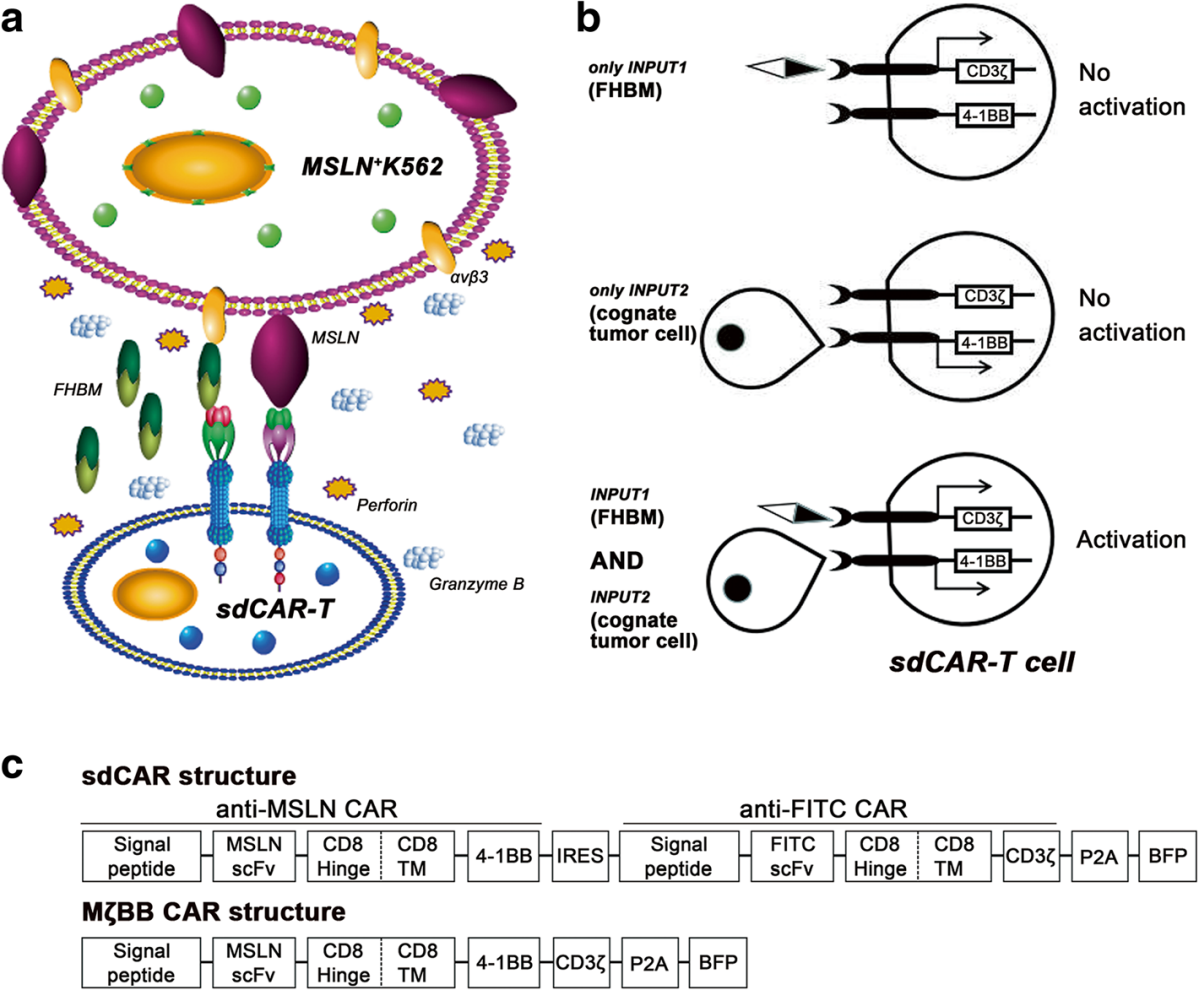
Schematic representation of sdCAR-engineered T cells. **a** Engineered T cells endowed with the sdCAR structure can eliminate MSLN^+^ K562 tumor cells in the presence of FHBM. Engineered T cells can release perforin and granzyme when they contact cognate target cells expressing both integrin αvβ3 and MSLN in the presence of a switch molecule (FHBM). Perforin can form a perforated tubular channel on the target cell membrane, resulting in the destruction of target cells. In addition, released granzyme rapidly enters the cytoplasm through perforin-dependent pores on the target cell membrane and then triggers caspase reactions that lead to target cell DNA degradation and tumor cell apoptosis. **b** The sdCAR design distributes key components (CD3ζ and 4-1BB elements) into two physically separate structures that can be conditionally controlled by a switch molecule and cognate tumor cells, respectively. The novel design involves an AND logic gate including the “cognate tumor cell + switch molecule” combinatorial inputs for T cell activation. Therefore, the sdCAR-engineered T cells should activate only when simultaneously exposed to the switch molecule (FHBM) and cognate tumor cells. **c** Structure of the plasmid used to transfect T cells. After transfection with this plasmid, effector cells expressed the novel sdCAR structure consisting of anti-MSLN scFv, anti-FITC scFv, two signal domains of 4-1BB and CD3ζ, and the fluorescence reporter protein BFP

To engineer an sdCAR that simultaneously interacts with both a tumor antigen and an exogenous molecule for cell activation and proliferation, a dual-receptor CAR model was designed based on the signal pathways of natural immune T cell receptor (TCR). sdCAR consisted of two receptors to control downstream signaling elements, including the immunoreceptor tyrosine-based activation motifs from the TCR/CD3ζ subunit and a co-stimulatory signal element 4-1BB (Fig. 1a). To avoid the transient cytotoxicity found in the first generation of CAR-T cells, the first receptor to control intracellular signaling element CD3ζ was designed with specificity for the switch molecule, FHBM. The switch molecule served as a priming factor and a prerequisite for the activation, proliferation, and cytotoxicity of sdCAR-engineered T cells. In addition, the other receptor that targeted tumor antigen MSLN regulated the intracellular co-stimulatory domain 4-1BB, which enhanced sdCAR-T cell proliferation and survival (Fig. 1b). The two parts of sdCAR were separated by an *IRES* (internal ribosome entry site) sequence, and each part contained an N-terminal CD8α signal peptide, which anchored it to the cellular membrane. In addition, each antigen-binding domain (single-chain variable fragment, scFv) was followed by a CD8 hinge sequence and a CD8 transmembrane element. Finally, to facilitate detection of sdCAR expression, a BFP was included downstream of the sdCAR structure by a P2A sequence (Fig. 1c).

### sdCAR expression on primary human T cells

**Fig. 2:**
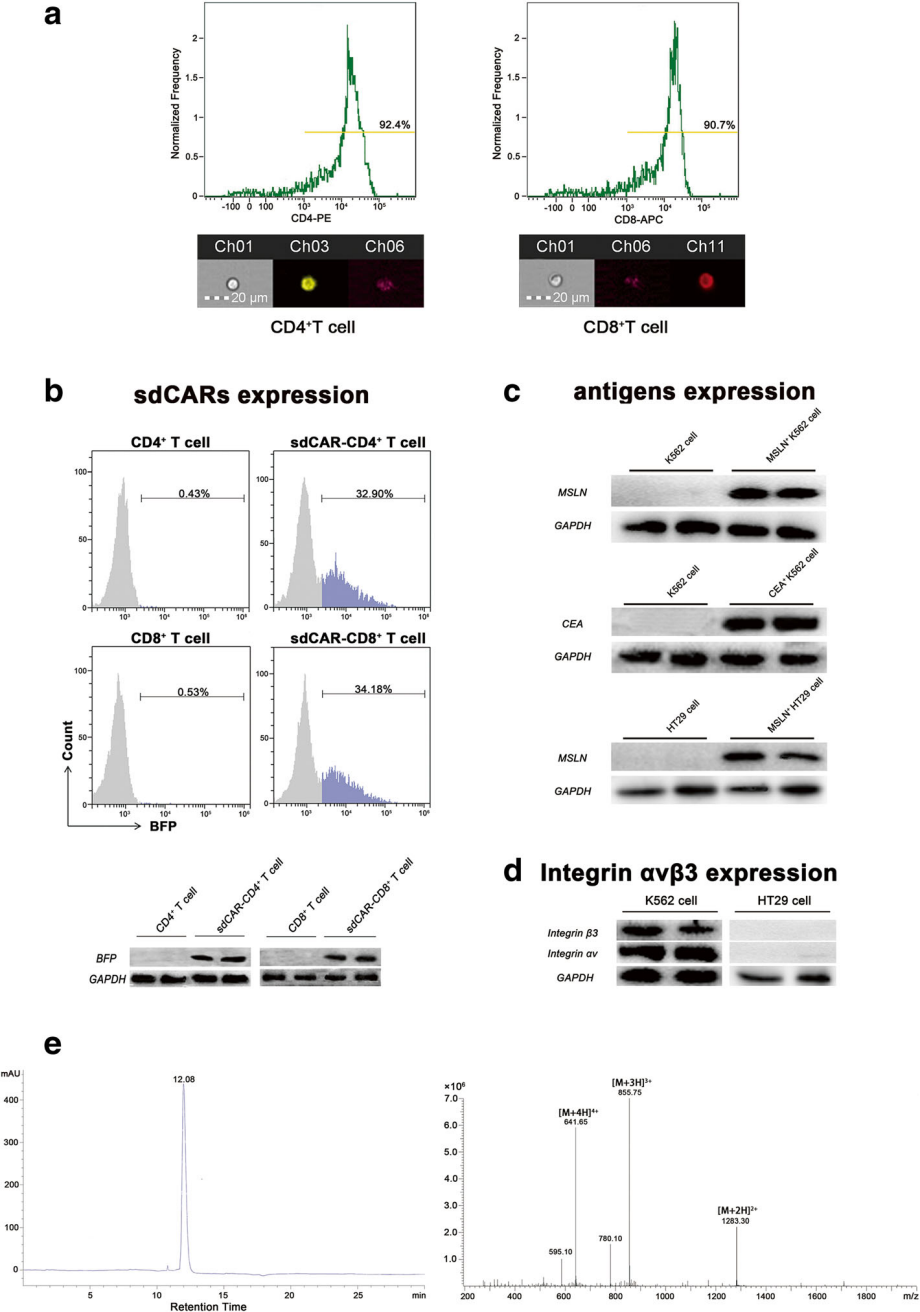
Construction and identification of effector cells, target cells, and bifunctional molecules. **a** Characterization of CD4^+^ T cells or CD8^+^ T cells enriched with the magnetic microbeads by flow cytometry. With anti-CD4-PE and anti-CD8-APC antibodies, the sorting efficiency of CD4^+^ T cells and CD8^+^ T cells was determined to be 92.4 and 90.7%, respectively (shown on the upper). At the single-cell level, our flow cytometry data indicated the size and morphology of cells attached to antibodies (shown on the lower). (Ch02 bright channel, Ch03 PE fluorescent channel, Ch11 APC fluorescent channel) **b** sdCAR-engineered T cells were successfully constructed by electroporation. We evaluated the transduction efficiency by flow cytometry with endogenous BFP expression to quantify fractions of sdCAR-CD4^+^ T cells and sdCAR-CD8^+^ T cells. **c** To assess sdCAR-T cell activity, K562 cells were lentivirally transduced to stably express human tumor antigens MSLN or CEA, respectively. Western blotting results confirmed the expression of cognate antigen (MSLN) on MSLN^+^ K562 cells (shown on the upper), non-cognate antigen (CEA) on CEA^+^ K562 cells (shown on the middle), and cognate antigen (MSLN) on MSLN^+^ HT29 cells (shown on the lower). We used glyceraldehyde 3-phosphate dehydrogenase (GAPDH) as an endogenous control. **d** Western blot results showed that the integrin αvβ3 was highly expressed on the surface of K562 cells (shown on the left). In contrast, HT29 cells did not express integrin αvβ3 (shown on the right). GAPDH was used as an endogenous control. **e** Analysis of site-specific FHBM conjugates after isolation and purification. Chromatography of obtained FHBM demonstrated that the final product was of high purity (> 98%) and can be used to regulate CAR-T activity (shown on the left). Characteristic ion peaks from mass spectrometry, such as [M+4H]^4+^, [M+3H]^3+^, and [M+2H]^2+^, showed that the molecular weight of FHBM was approximately 2565 and the ratio of FITC to HM-3 in FHBM conjugate was about 1.97 (shown on the right)

Blood from healthy donors was used to isolate fresh peripheral blood mononuclear cells, which were further sorted into CD4^+^ T cells or CD8^+^ T cells (Fig. 2a). Primary T cells were engineered with a dual receptor that features the FITC antigen receptor and the MSLN antigen receptor. We generated a new class of CAR whose small molecule-induced phosphorylation of the CD3ζ signaling domain was necessary but not sufficient for effector T cell activation. Next, we have verified the accurate transcription of the sdCAR gene in engineered CAR-T cells by sequencing. As a result, the sdCAR fusion gene was present in the genomes of sdCAR-engineered T cells (Additional file 1: Figure S1). In addition, we tested whether the sdCAR structure was presented on the surface of the engineered T cells. Using BFP fluorescence, the sdCAR expression was qualitatively and quantitatively characterized by flow cytometry and Western blot assays (Fig. 2b). The transduction efficiencies of sdCAR-CD4^+^ T and sdCAR-CD8^+^ T cells were 32.90 and 34.18%, respectively.

### Design of target cells and the bifunctional molecule FHBM

To investigate the specificity and cytotoxicity of sdCAR-T cells, we subcloned the cognate target antigen (MSLN) gene into a lentiviral vector containing green fluorescent protein (GFP). First, we confirmed that there was no expression of MSLN or CEA antigen on wild-type K562 cells (Fig. 2c, Additional file 1: Figure S2a). We used lentiviral particles derived from HEK293T cells to infect K562 cells and eventually obtained cognate target cells (MSLN^+^ K562) by puromycin selection. In our experiments, we also generated non-cognate target cells, CEA-positive K562 cells, to assess cognate target cell killing in vitro and in vivo (Additional file 1: Figure S2b). Additionally, K562 cells highly express integrin αvβ3, which can be targeted specifically by the antitumor peptide HM-3 part of FHBM (Fig. 2d). In our study, we also selected another tumor cell line, HT29, which lacks integrin αvβ3 expression, as a negative control to verify the specificity of the novel switch molecule. After lentivirus transfection, HT29 cells overexpressed the cognate tumor antigen MSLN (Fig. 2c, Additional file 1: Figure S2c).

Following the manufacturer’s instructions, we used FITC coupled with HM-3 peptide (FHBM) as a safe switch to control the activity of FITC-specific CAR-T cells. The FITC/HM-3 molar ratio was defined as the ratio of FITC to HM-3 peptide in the conjugate. We performed purity and structure analyses of isolated FHBM with chromatography and mass spectrometry, respectively (Fig. 2e). After purification, the obtained product was more than 98.83% pure (*R*_t_ = 12.08 min), which can be used as an efficacious switch to regulate sdCAR-T cell activity in the follow-up experiment. From the mass spectrometry data, the molecular weight of FHBM was 2565. Based on the molecular weights of FITC (389) and HM-3 (1797), we can infer that the ratio of FITC to HM-3 in the final conjugate was 1.97. Finally, the obtained compound had a concentration of 5.42 μM and an FITC/HM-3 molar ratio of 1.98 by spectrophotometry, which are in line with the mass spectrometry data.

### Activation of human CD4^+^ T cells engineered with sdCAR requires the switch molecule and the cognate tumor cells

**Fig. 3:**
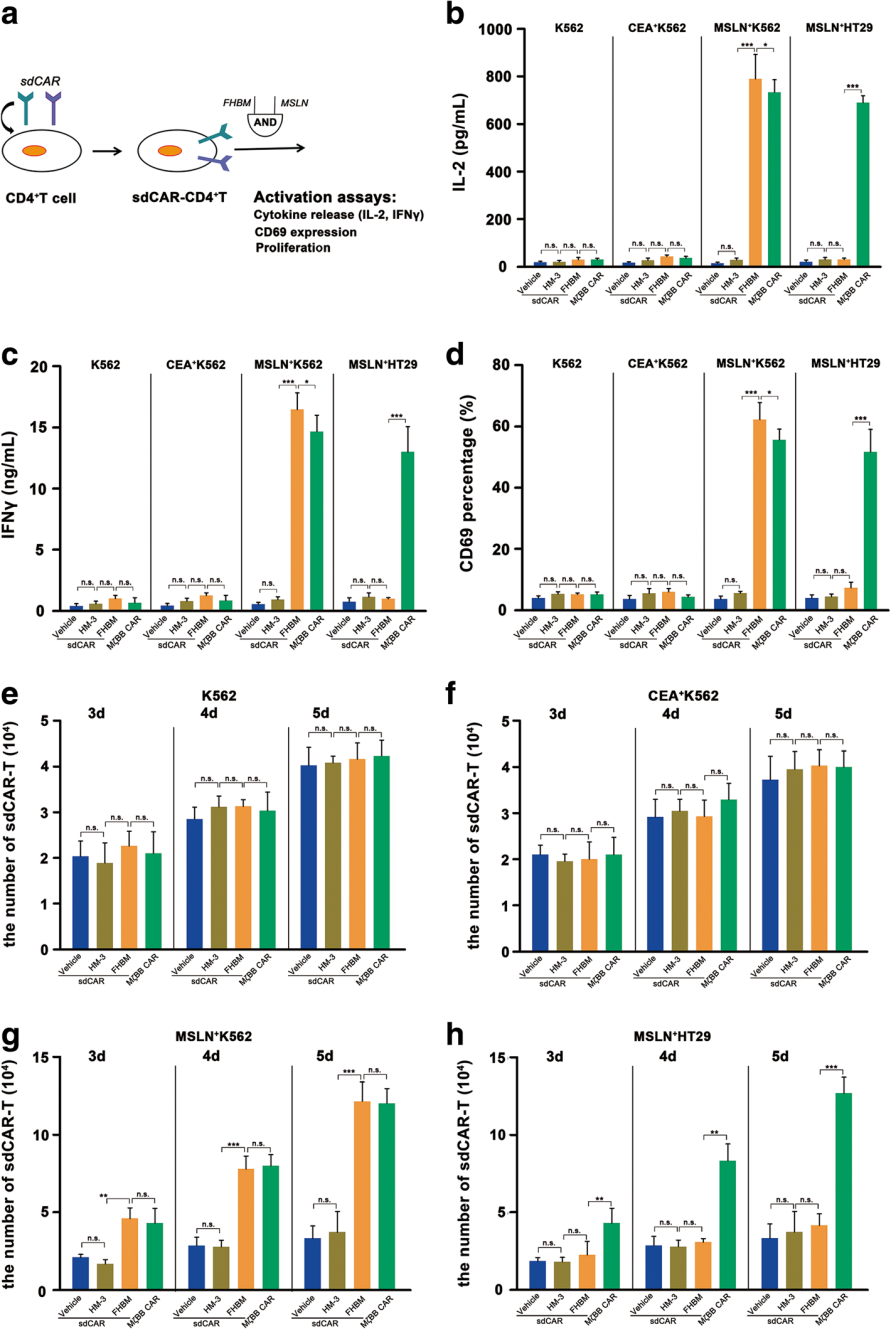
Switch molecule-controlled activation of CD4^+^ T cells engineered with sdCAR. **a** We engineered primary human CD4^+^ T cells derived from fresh human peripheral blood with sdCARs by electroporation and evaluated the cells by activation assays with an “AND logic gate” strategy. **b**, **c** We quantified production of IL-2 and IFNγ by enzyme-linked immunosorbent assay. The cytokines were produced only when sdCAR-T cells were exposed to cognate tumor cells (MSLN^+^ K562) in the presence of FHBM but not when exposed to non-cognate tumor cells (K562 or CEA^+^ K562). For MSLN^+^ HT29 cells, high levels of cytokines were found only in MζBB CAR-T cells. (*n* = 3, error bars denote standard deviation.) **d** Monitoring T cell activation by CD69 expression. CD69 was expressed on sdCAR-T cells in the presence of both MSLN^+^ K562 cells and a switch molecule (FHBM) and also was expressed on MζBB CAR-T cells. For MSLN^+^ HT29 cells, only MζBB CAR-T cells had significant CD69 expression. (*n* = 3, error bars denote standard deviation.) **e**–**h** FHBM and cognate tumor cell-dependent T cell proliferation. As sdCAR-T cells express a fluorescence reporter protein (BFP), we quantified the number of activated cells by flow cytometry after 3, 4, or 5 days of incubation. For cognate tumor cells (K562 or CEA^+^ K562), sdCAR-T cells and MSLN-specific CAR-T cells had no significant proliferation. sdCAR-T cell proliferation was regulated by FHBM in the presence of MSLN^+^ K562 cells. A similar degree of T cell proliferation was found in MζBB CAR-T cells. For HT29 tumor cells, only MSLN-specific CAR-T cells had strong proliferation. (*n* = 3, error bars denote standard deviation)

Activation responses of sdCAR-engineered CD4^+^ T cells, including IL-2 production, IFNγ production, and CD69 expression were detected in the presence of MSLN^+^ K562 cells and switch molecules. Theoretically, the tumor antigen and the switch molecule were required for sdCAR-T cell activation based on the dual signaling pathway of the engineered T cells (Fig. 3a).

As shown in Fig. 3b, c, when co-cultured with K562 cells or CEA^+^ K562 cells, sdCAR-T cells secreted base levels of IL-2 or IFNγ whether treated with PBS, HM-3, or FHBM, which was similar to MζBB CAR-T cells. sdCAR-T cells co-cultured with MSLN^+^ K562 cells also secreted base levels of IL-2 or IFNγ in the presence of PBS or HM-3. The levels of these cytokines released from sdCAR-T cells substantially increased, however, when co-cultured with MSLN^+^ K562 cells in the presence of FHBM at a final concentration of 100 pM, which was significantly higher than MζBB CAR-T cell activity. For MSLN^+^ HT29 cells without integrin αvβ3 expression, only MζBB CAR-T cells secreted significant levels of cytokines. These results demonstrated that FHBM specifically targeted the FITC receptor expressed on sdCAR-T cells and effectively activated them in the presence of MSLN^+^ K562 cells. Therefore, FHBM can be developed as an efficacious switch to control the activity of sdCAR-T cells, resulting in the controllable activation of T cells and then the destruction of target cells. In addition, there was no T cell activation for the non-cognate tumor cell line (K562 cell or CEA^+^ K562 cell) in contrast to the significant activation for cognate tumor cells (MSLN^+^ K562), thereby showing specific targeting activity of sdCAR-T cells.

Similarly, for sdCAR-T cells, a remarkable upregulation of CD69, an activation marker presented on T cell surfaces, was observed only in the presence of cognate tumor cells (MSLN^+^ K562) and the switch molecule FHBM. T lymphocytes expressing MζBB CARs displayed a high level of CD69 expression while in contact with cognate tumor cells, whereas sdCAR described here required an additional switch molecule for high expression of CD69, allowing for control of T cell activity (Fig. 3d, Additional file 1: Figure S3).

### Proliferation of sdCAR-engineered human CD4^+^ T cells requires the switch molecule and cognate tumor cells

We next evaluated whether the proliferation of CD4^+^ T cells engineered with sdCAR could be controlled by the switch molecule and MSLN^+^ K562 cells. Using flow cytometry assays, the percentage of sdCAR-modified CD4^+^ T cells can be determined by measuring the constitutive expression of BFP inserted downstream of sdCAR. For the non-cognate target cells (K562 cells or CEA^+^ K562 cells), sdCAR-T cell had no significant proliferation (Fig. 3e, f). In the presence of MSLN^+^ K562 cells, sdCAR-T cell proliferation was regulated by the switch molecule FHBM. A similar degree of T cell proliferation was characterized in MζBB CAR-T cells only in the presence of MSLN^+^ K562 cells (Fig. 3g). For HT29 cells expressing MSLNs, no sdCAR-T cells showed proliferation whether treated with PBS, HM-3, or FHBM; however, MSLN-specific CAR-T cells had strong proliferation (Fig. 3h). Proliferation allowed for amplification of T cell function, but excessive proliferation can initiate some systemic and severe toxicities, such as CRS and “on-target, off-tumor” toxicity. Therefore, the new molecule FHBM acts as a switch to effectively regulate sdCAR-T cell proliferation, avoiding some of the side effects derived from the overactivation of sdCAR-T cells.

### Dual-gated antitumor cytotoxicity of sdCAR-engineered CD8^+^ T cells

**Fig. 4:**
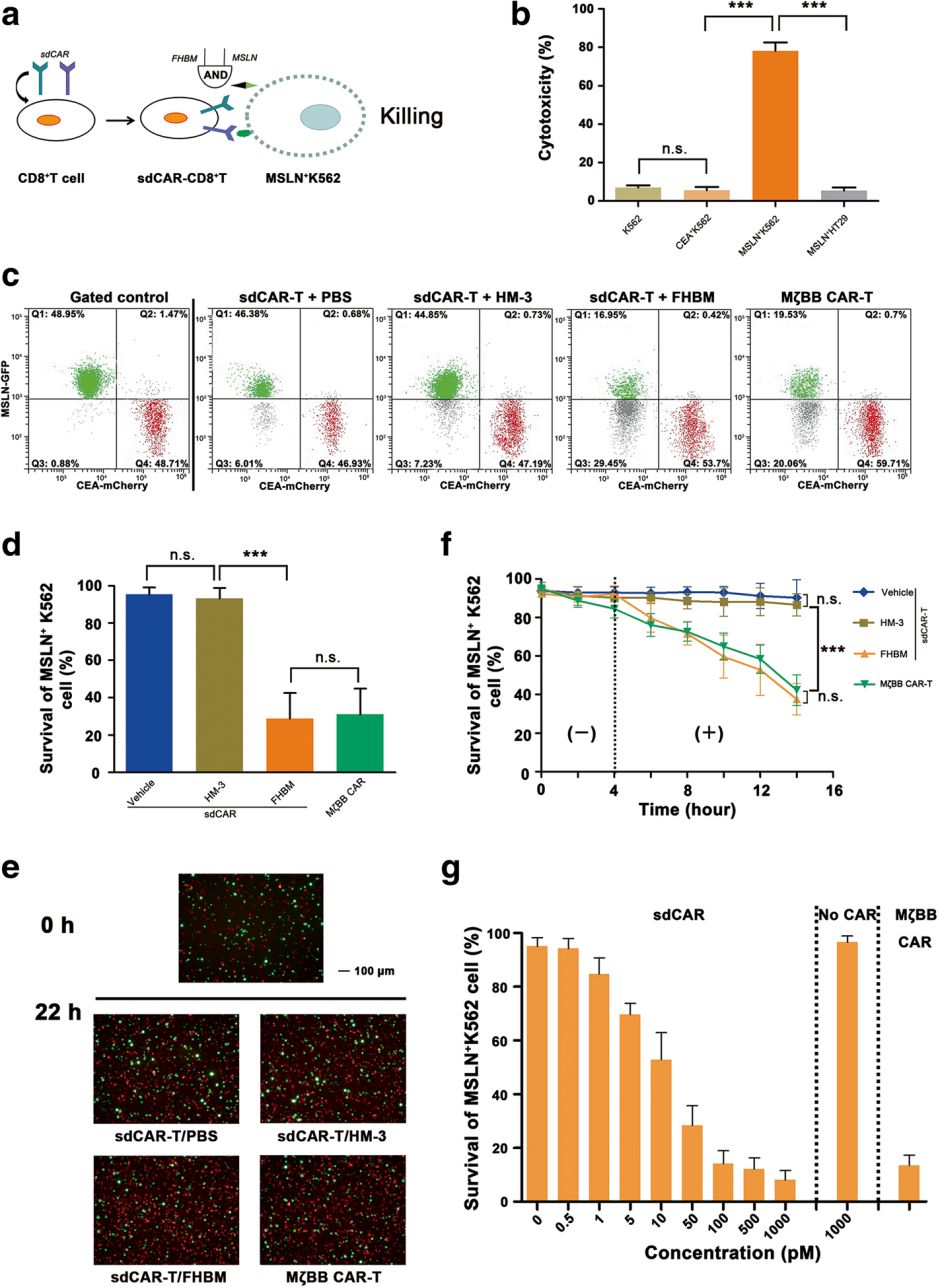
sdCAR-engineered CD8^+^ T cells yield antigen-specific and titratable killing of cognate target cells in vitro. **a** sdCAR-engineered CD8^+^ T cells eradicated cognate target cells expressing MSLN antigen and integrin αvβ3 with an “AND logic gate” strategy. **b** Specific cytotoxicity of sdCAR-engineered T cells. sdCAR-T cells combined with FHBM have significant cytotoxicity for MSLN^+^ K562 cells. **c** Representative flow cytometry data for cytotoxicity of engineered-CD8^+^ T cells. T cells were incubated with a mixture of cognate target cells (GFP^+^) and non-cognate target cells (mCherry^+^). After a 22-h incubation, we quantified the abundance of both surviving cognate and non-cognate tumor cells. The normalized percentage of surviving cognate target cells is expressed as the percentage of MSLN^+^ K562 cells (Q1) divided by that of CEA^+^ K562 cells (Q4). **d** Cytotoxicity mediated by sdCAR-CD8^+^ T cells in a 22-h experiment. The lower surviving percentage of MSLN^+^ K562 cells indicated a significant degree of cognate target cell killing by sdCAR T cells only upon the addition of FHBM. (*n* = 3, error bars denote standard deviation.) **e** Representative fluorescence images of target cells. After 22 h of interaction, we observed mixtures of cognate and non-cognate target cells incubated with engineered-CD8^+^ T cells by fluorescence microscopy. **f** Time course of cognate target cell killing by sdCAR-T cells. The sdCAR-T cell cytotoxicity was not monitored in the absence of switch molecules for the first 4 h (−). However, sdCAR-CD8^+^ T cells exerted high cytotoxicity for cognate target cells only in the presence of FHBM (+). (*n* = 3, error bars denote standard deviation.) **g** Effect of the switch molecule dose on the cytolytic capacities of sdCAR-T cells. The level of cognate target cell killing was correlated with concentrations of FHBM. When the concentration of the switch molecule was increased to more than 100 pM, there was no further increase of T cell cytotoxicity. (*n* = 3, error bars denote standard deviation)

Specific apoptosis of tumor cells expressing target antigens is a hallmark of CAR-T cell behavior, which is caused by the cytotoxicity of CD8^+^ T cells endowed with a CAR specific for a cognate antigen (Fig. 4a). Conventional CAR-T cell-mediated target cell killing has a short time window and has produced a significant level of cytokines in some clinical trials. First, we have verified that CD8^+^ T cells engineered with sdCAR have specific cytotoxicity for the cognate tumor cell (MSLN^+^ K562) in the presence of FHBM (Fig. 4b). In the present study, we coupled K562 cells expressing MSLN or CEA with the selective expression of a distinct fluorescent protein, such as GFP or mCherry, to determine the cytotoxicity of sdCAR-T cells by flow cytometry (Fig. 4c). Results showed that no significant cell-mediated cytotoxicity was observed in sdCAR-T cells with treatment of HM-3 or PBS; however, the efficient killing of cognate target cells occurred when using FHBM. The degree of cognate tumor cell killing mediated by sdCAR-T cells in the presence of FHBM matched the level observed with MζBB CAR-T cells (Fig. 4d). In addition, we observed the mixture of cognate and non-cognate target cells by fluorescence microscopy after 22 h of incubation with sdCAR-T cells. Images showed that the percentage of MSLN^+^ K562 cells in the mixture gradually reduced over time only using FHBM. Interestingly, we also observed similar cognate tumor cell killing in the presence of MζBB CAR-T cells (Fig. 4e, Additional file 1: Figure S4). Collectively, we confirmed that the cytotoxicity of sdCAR-T cells was effectively controlled with the simultaneous presence of exogenous molecules and cognate tumor cells. Therefore, to kill tumor cells expressing MSLN and integrin αvβ3, FHBM can be used as a switch to tune the cytotoxicity of CD8^+^ T cells expressing an sdCAR specific for MSLN and the FITC part of FHBM.

We next investigated whether the sdCAR framework provided temporal control of T cell cytotoxicity. During a 14-h experiment, CD8^+^ T cells expressing the sdCAR structure did not have specific cytotoxicity for cognate target cells until the exogenous addition of FHBM (Fig. 4f). In vitro cytotoxicity experiments showed that MSLN^+^ K562 cells were killed by sdCAR-expressing CD8^+^ T cells in an FHBM-dependent manner. We next determined whether the sdCAR-T cell cytotoxicity could be immediately terminated by FHBM removal and then resumed after the re-addition of FHBM. Our proof-of-principle experiment consisted of three stages to implement the presence-absence-presence of FHBM in co-cultures of sdCAR-T and MSLN^+^ K562 cells (Additional file 1: Figure S5a). The data showed that sdCAR-T cell cytotoxicity was detected only in the first and third stages of the experiment; therefore, FHBM provided a flexible platform for temporal control over CAR-T cell action (Additional file 1: Figure S5b). The cytotoxicity of T cells expressing this dual-receptor CAR was strictly dependent on the exogenous molecule FHBM in addition to cognate tumor cells expressing MSLN and integrin αvβ3.

Next, we measured whether the cytolytic capacities of the engineered CD8^+^ T cells depended on the dose of FHBM. The results revealed that the percentage of surviving cognate target cells decreased with an increase of FHBM concentration (Fig. 4g). The killing of cognate target cells with lower concentrations of FHBM (≤ 0.5 pM) was similar to the killing levels of wild-type T cells without CAR engineering. In contrast, at higher concentrations (≥ 100 pM), sdCAR-modified CD8^+^ T cells yielded a similar antitumor response with a less than 20% survival rate of target cells because the FITC receptor of sdCAR has been saturated. In addition, sdCAR-T cells in the presence of 100 pM FHBM had a similar cytotoxicity compared with that of conventional CAR-T cells (MζBB CAR-T cells). Therefore, the new CAR structure with a switchable receptor functions as a dial for titratable control over the magnitude of apoptosis in the target cell population.

### In vivo cognate tumor cell killing in the presence of a switch molecule

**Fig. 5:**
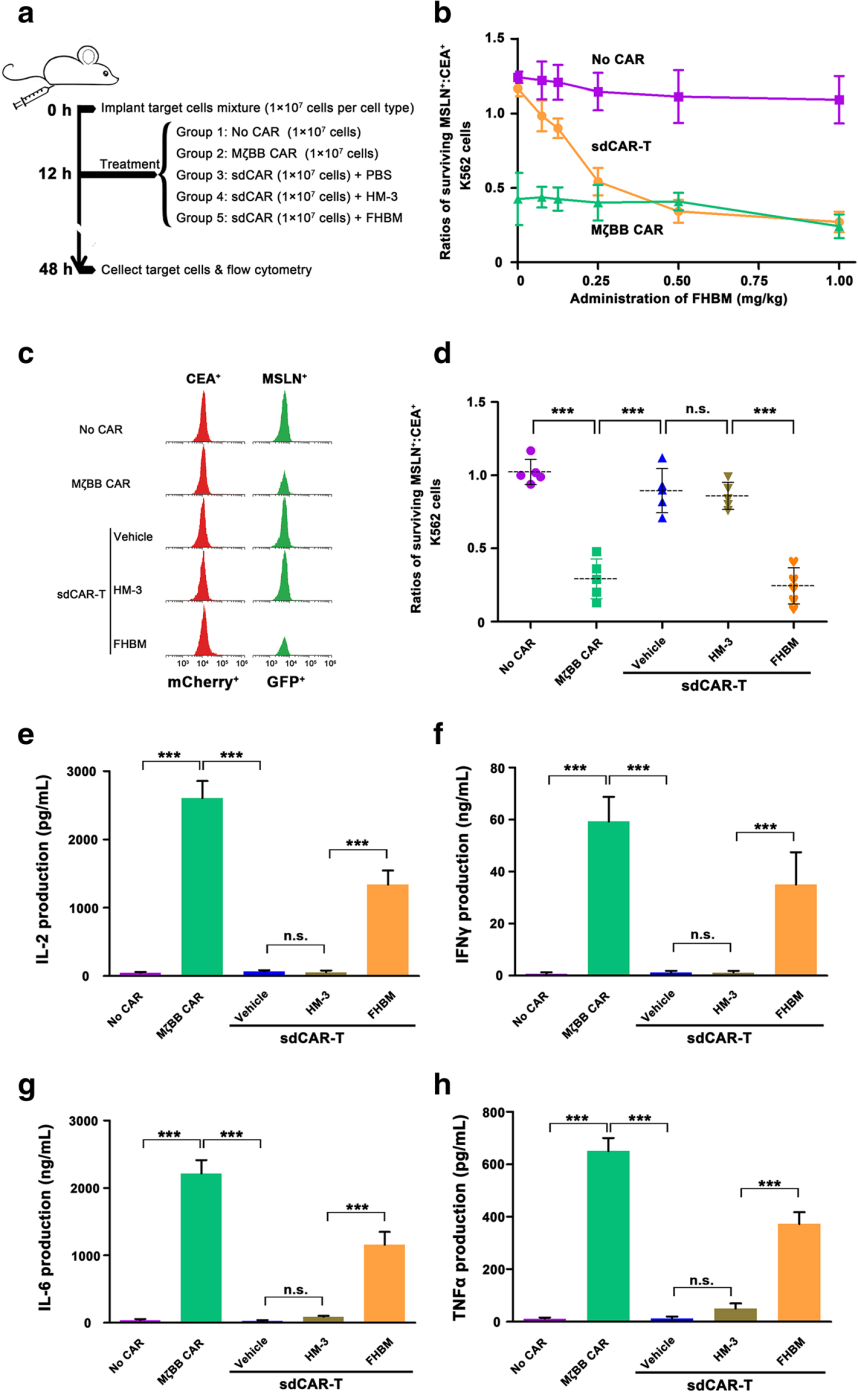
In vivo cytotoxic effect of sdCAR-T cells toward the cognate tumor cells. **a** Schematic of the mouse treatment strategy used in the in vivo experiment. Matched MSLN^+^/CEA^+^ K562 cells described previously were injected into nude mice intraperitoneally (i.p.). Effector cells, switch molecules, or PBS were injected i.p. at the indicated time point. After 48 h, both target cells were recovered from peritoneal lavage and quantified by flow cytometry. **b** Various concentrations of FHBM were added to mice. The degree of cytotoxicity of sdCAR-T cells together with saturated FHBM (0.5 mg/kg) matched the level observed with the MζBB CAR-T cells that triggered significant cytotoxicity for cognate tumor cells. **c** At the end of the experiment, we quantitatively analyzed the remaining cognate and non-cognate tumor cells by flow cytometry, respectively. sdCAR-T cells eliminated the cognate tumor cells in mice treated with FHBM, comparable to the cytotoxicity of MζBB CAR-T cells. **d** Ratios of surviving MSLN^+^ K562:CEA^+^ K562 in each sample. Cognate target cells were killed by sdCAR-T cells only in the presence of FHBM. We observed similar results for MζBB CAR-T cells. (*n* = 5, error bars denote standard deviation.) **e**–**h** The sdCAR-T cells produced lower levels of cytokines. During the experiment, sdCAR-T cells released IL-2 (~ 1300 pg/mL), IFNγ (~ 30 ng/mL), IL-6 (~ 1160 ng/mL), and TNFα (~ 380 pg/mL) only in the presence of FHBM. MζBB CAR-T cells always released high levels of cytokines, including ~ 2600 pg/mL of IL-2, ~ 60 ng/mL of IFNγ, ~ 2216 ng/mL of IL-6, and ~ 647 pg/mL of TNFα. (*n* = 5, error bars denote standard deviation)

We determined whether sdCAR-engineered primary T cells had controllable cytotoxicity in a switch-dependent manner in a mouse xenograft model. A representative schematic of in vivo experiments was shown in Fig. 5a. First, we performed on sdCAR-T cells together with various concentrations of FHBM in mice and found that the tumor cell killing at 0.5 mg/kg of FHBM rivaled that obtained with T cells expressing MζBB CAR (Fig. 5b). According to the experiment results, a significant elimination of MSLN^+^ K562 cells was obtained in mice injected with sdCAR-T cells and FHBM, and a similar cell killing was manifested in mice with the treatment of MζBB CAR-T cells. In contrast, there was no cytotoxicity of sdCAR-T cells for cognate antigen expressing tumor cells in mice with the addition of HM-3 or PBS, a result similar with that of the intact T cells without the CAR structure (Fig. 5c, d, Additional file 1: Figure S6). In addition, we examined cytokine changes over time, including those of IL-2, IFNγ, IL-6, and TNFα in mice after injection of modified T cells (Additional file 1: Figure S7). The levels of these cytokines obtained from MζBB CAR-T cells were twice as high as those from sdCAR-T cells in the presence of FHBM (Fig. 5e–h). In addition, by pharmacokinetic analysis, FHBM had a plasma half-life of approximately 20.06 h in mice (Additional file 1: Figure S8). This property resulted in the highest levels of cytokines released from activated T cells in mice at 20 h and then gradually diminished. Therefore, the CRS toxicity derived from the excessive cytotoxicity of conventional CAR-T cells was mitigated by using sdCAR-T cells together with the switch molecule FHBM.

Next, we verified whether sdCAR-engineered T cells combined with a bifunctional molecule can achieve positive responses against solid tumor expressing integrin αvβ3 and MSLN in xenograft. First, we demonstrated that the pancreatic cancer cell line AsPC-1 highly expressed the integrin αvβ3 and MSLN, and the target cells were genetically engineered to express the red fluorescent protein (RFP) (Additional file 1: Figure S9a-S9c). Consistent with the previous study, mice treated with sdCAR-T cells and FHBM (0.5 mg/kg) achieved rapid tumor clearance at day 20 (after sdCAR-T cell infusion), which was similar to that of mice treated with MζBB CAR-T cells (Additional file 1: Figure S9d).

In agreement with our in vitro results, the combination of FHBM and sdCAR-T cells resulted in high killing efficiency of cognate target cells expressing integrin αvβ3 and MSLN in vivo. These results demonstrated that the activity of CAR-T cells was significantly enhanced through both the dual-receptor CAR and a bifunctional molecule. Finally, we confirmed that the sdCAR-T cell cytotoxicity was effectively controlled with two combined inputs in vivo, a cognate tumor cell and a switch molecule, thereby mitigating CRS and “on-target, off-tumor” toxicity.

## Discussion

With advances in tumor biology and immunology, the adoptive transfer of T cells expressing CARs has become a new approach for cancer treatment in recent years [7]. To achieve specificity and controllable cytotoxicity of CAR-T cells, several strategies have been described that use either negative regulation or positive regulation of the T cell signaling pathway. In the present study, the activity of engineered T cells endowed with CARs was significantly improved through the simultaneous actions of a dual-receptor CAR and a bifunctional molecule. For the first time, we proposed a dual-receptor CAR specific for FITC and MSLN, and its cytotoxicity was effectively controlled with the use of two combined inputs, a switch molecule and cognate tumor cells expressing integrin αvβ3 and MSLN, thereby enhancing the safety of CAR-T cell therapy. In addition, sdCAR-T cells with FHBM cleared MSLN^+^ K562 cells with lower levels of cytokine release relative to MζBB CAR-T cells in an in vivo experiment, thereby mitigating CRS toxicity.

In our results, FHBM as a switch molecule is required for sdCAR-T cell activity including activation, proliferation, and cytotoxicity. In addition, cognate tumor cells expressing MSLN and integrin αvβ3 are required for sdCAR-T cell function. Compared with the first generation of CAR-T cells, sdCAR-T cells designed here are not activated in the presence of FHBM and tumor cells that express integrin αvβ3 and do not express MSLN (such as CEA^+^ K562 cells). Theoretically, the FITC receptor expressed on T cell surfaces has a weak affinity for FHBM, which triggers the phosphorylation of the intracellular CD3ζ signal domain yet is not sufficient to initiate T cell activation [17, 28–30]. Therefore, sdCAR-T cells were inactivated with only the use of FHBM, potentially avoiding the anaphylaxis observed in the FITC-folate clinical trial described previously [31]. Interestingly, in the presence of cognate tumor cells and FHBM, sdCAR-T cells were synergistically activated because of the phosphorylation of both the CD3ζ and 4-1BB signal domains. Thus, the sdCAR-T cell activity was strictly dependent on the switch molecule and cognate tumor cells.

**Fig. 6:**
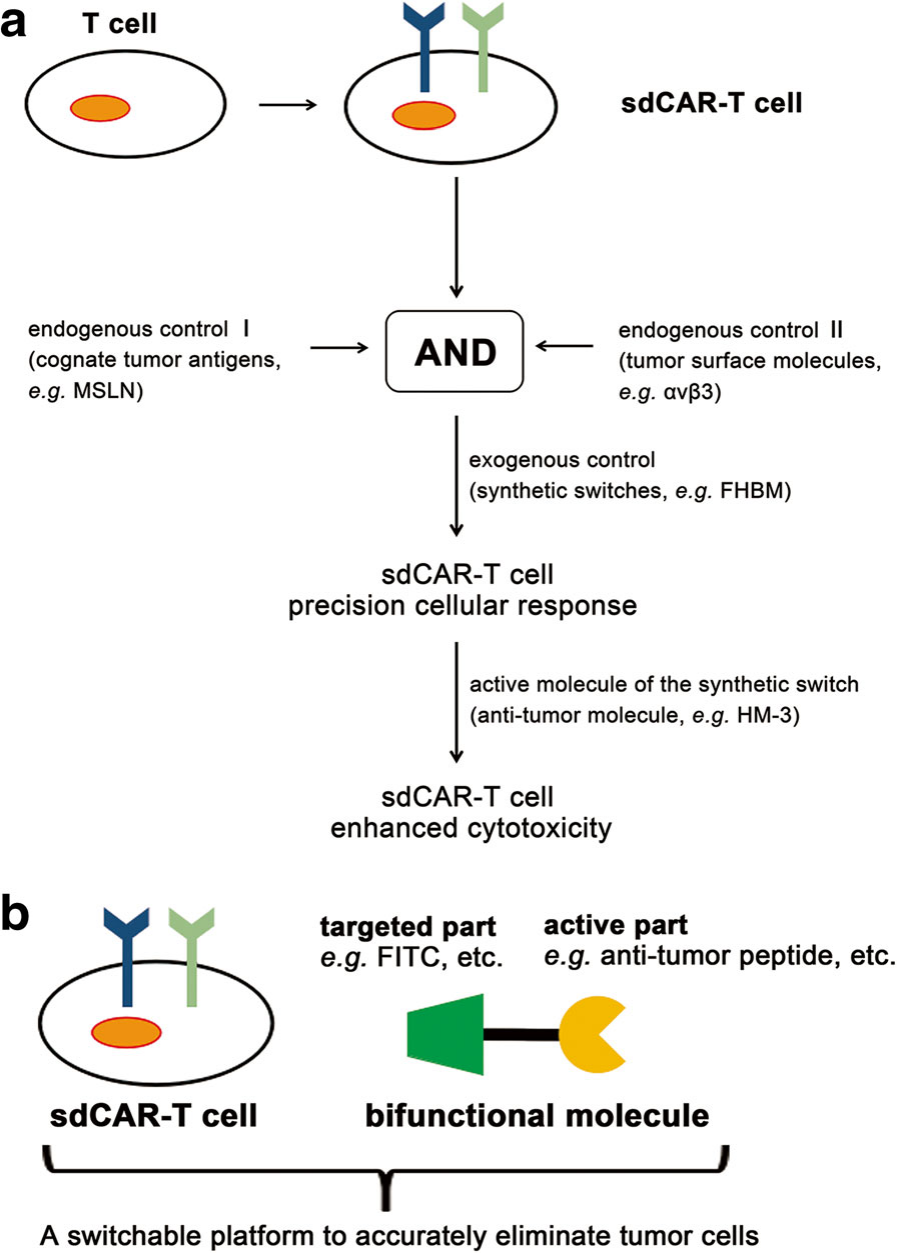
Immunotherapeutic strategy for engineering T cells that integrate endogenous and exogenous controls. **a** sdCAR framework provides an important tool for developing the next generation of therapeutic T cells that can be precisely controlled. In this system, modified T cells have potent therapeutic effects upon simultaneously recognizing an endogenous control (e.g., cognate tumor antigens and other molecules of interest) and an exogenous control (e.g., switch molecules) in a temporally and spatially regulated manner, thereby producing a precision therapeutic response. As target cells express molecules of interest (e.g., αvβ3) that target the active part of a bifunctional molecule (e.g., the HM-3 part of FHBM), the target cell killing of this system is further enhanced by the active part suppressing tumor growth. **b** This synthetic combinatorial control system can provide a switchable platform for specific elimination of cognate tumor cells. For the bifunctional molecule, FITC coupled with antitumor peptide or remodeling factor against the tumor immunosuppressive microenvironment is compatible with the switchable capability of FHBM to regulate the activity of sdCAR-T cells

In general, the type of cellular regulation described here integrates a cognate tumor antigen (e.g., MSLN) with a tumor surface molecule (e.g., αvβ3) presented on target cells (Fig. 6a). We designed a novel bifunctional molecule by connecting FITC to the N-terminus of the integrin αvβ3-targeting peptide HM-3, which by itself has anti-angiogenic and antitumor activity. Results showed that the fusion switch molecule regulated sdCAR-T cell activity and did not interfere with FITC targeting of sdCAR-T cells. Hence, the development of a universal sdCAR forms an effective platform for treating MSLN-positive tumors expressing integrin αvβ3 in the presence of an FITC-based bifunctional molecule (FHBM). FHBM can be used not only as a switch to regulate the activity of sdCAR-T cells but also as an antitumor molecule to inhibit tumor growth by anti-angiogenesis, providing an important platform for CAR-T cell therapy for the future treatment of solid tumors.

We envision that our sdCAR structure combined with a bifunctional molecule can achieve positive responses against solid tumors in spite of the blunt immune-surveillance, including some immune suppressor cells, cytokines, and physical barriers around tumor tissues [32–34]. In our opinion, the bifunctional molecule containing both a targeted part specific for sdCAR-T cells and an active part with the ability to improve the tumor microenvironment serves as a “switch” to control the state of sdCAR-T cells and a “scavenger” that clears immunosuppressive factors or physical barriers around tumor tissues (Fig. 6b). Adoptive cell therapies involving sdCAR-T cells and a bifunctional molecule are thought to have a major effect upon at least one of these factors described above, thereby producing potential benefits in patients with refractory and relapsed malignancies. In conclusion, our sdCAR-T cell strategy making use of a bifunctional switch-molecule program T cells with a significant specificity and controllable cytotoxicity. The time- and dose-dependent regulation of sdCAR-T cell activity advances adoptive cell therapy using genetically modified T cells one step further toward precision medicine.

## Conclusions

Results from this observational study indicate that a new switch molecule FHBM can accurately regulate the switchable dual-receptor CAR-T (sdCAR-T) cell activity in a time- and dose-dependent manner and sdCAR-T cells exert significant antitumor activity while releasing lower levels of cytokines for the cognate tumor cells expressing both MSLN and integrin αvβ3. Therefore, the combination therapy using sdCAR-T cells and FHBM is becoming increasingly prospective in cancer immunotherapy.

## Additional file

Additional file 1: **Figure S1.** Identification of sdCAR structure gene in sdCAR-engineered T cells. **Figure S2.** Detection of cognate and non-cognate antigen expression on K562 cells or HT29 cells. **Figure S3.** Expression levels of CD69 on activated CAR-T cells. **Figure S4.** Representative fluorescence images of cognate antigen and non-cognate target cells after a 22-h incubation with sdCAR-T cells or MζBB CAR-T cells. **Figure S5.** sdCAR-T cell function was strictly dependent on FHBM. **Figure S6.** Schematic representation of flow cytometry-based target cell killing assay in vivo. **Figure S7.** The cytokine levels over time in mice after injection of modified-T cells. **Figure S8.** Calculate the value of the half-life of FHBM. **Figure S9.** sdCAR-T cell cytotoxicity for solid tumor in xenograft. (DOCX 20379 kb)

## Abbreviations

APC: Allophycocyanin
BFP: Blue fluorescent protein
CAR: Chimeric antigen receptor
CEA: Carcinoembryonic antigen
CRS: Cytokine release syndrome
FBS: Fetal bovine serum
FHBM: FITC-HM-3 bifunctional molecule
GFP: Green fluorescent protein
IFNγ: Interferon γ
IL-2: Interleukin-2
IL-6: Interleukin-6
IRES: Internal ribosome entry site
MSLN: Mesothelin
PBMCs: Peripheral blood mononuclear cells
PBS: Phosphate buffer saline
PE: Phycoerythrin
RFP: Red fluorescent protein
scFv: Single-chain variable fragment
sdCAR-T: Switchable dual-receptor CAR-T
TCR: T cell receptor
TNFα: Tumor necrosis factor α
