# Supplementary material for: Accurate control of dual-receptor-engineered T cell activity through a bifunctional anti-angiogenic peptide
Source: J Hematol Oncol. 2018 Mar 20;11:44. doi: 10.1186/s13045-018-0591-7 (PMC5859748; doi:10.1186/s13045-018-0591-7)
Supplement: Supplementary file 1 — Figure S1. Identification of sdCAR structure gene in sdCAR-engineered T cells. Figure S2. Detection of cognate and non-cognate antigen expression on K562 cells or HT29 cells. Figure S3. Expression levels of CD69 on activated CAR-T cells. Figure S4. Representative fluorescence images of cognate antigen and non-cognate target cells after a 22-h incubation with sdCAR-T cells or MζBB CAR-T cells. Figure S5. sdCAR-T cell function was strictly dependent on FHBM. Figure S6. Schematic representation of flow cytometry-based target cell killing assay in vivo. Figure S7. The cytokine levels over time in mice after injection of modified-T cells. Figure S8. Calculate the value of the half-life of FHBM. Figure S9. sdCAR-T cell cytotoxicity for solid tumor in xenograft. (DOCX 20379 kb) [file 13045_2018_591_MOESM1_ESM.docx]

**Additional file 1**

**Accurate control of dual receptor-engineered T-cell activity through a bifunctional antiangiogenic peptide**

Erhao Zhang^1^, Jieyi Gu^1^, Jianpeng Xue^1,2^, Chenyu Lin^1^, Chen Liu^1^, Mengwei Li^1^, Jingchao Hao^1,3^, Sarra Setrerrahmane^1^, Xiaowei Chi^1^, Weiyan Qi^1,2^, Jialiang Hu^1,2,*^ and Hanmei Xu^1,2,4,*^

***Correspondence:** Jialiang Hu, E-mail:[jialiang_hu51@aliyun.com;](mailto:jialiang_hu51@aliyun.com;) Hanmei Xu, E-mail: 13913925346@126.com

*Shared senior authorship

**
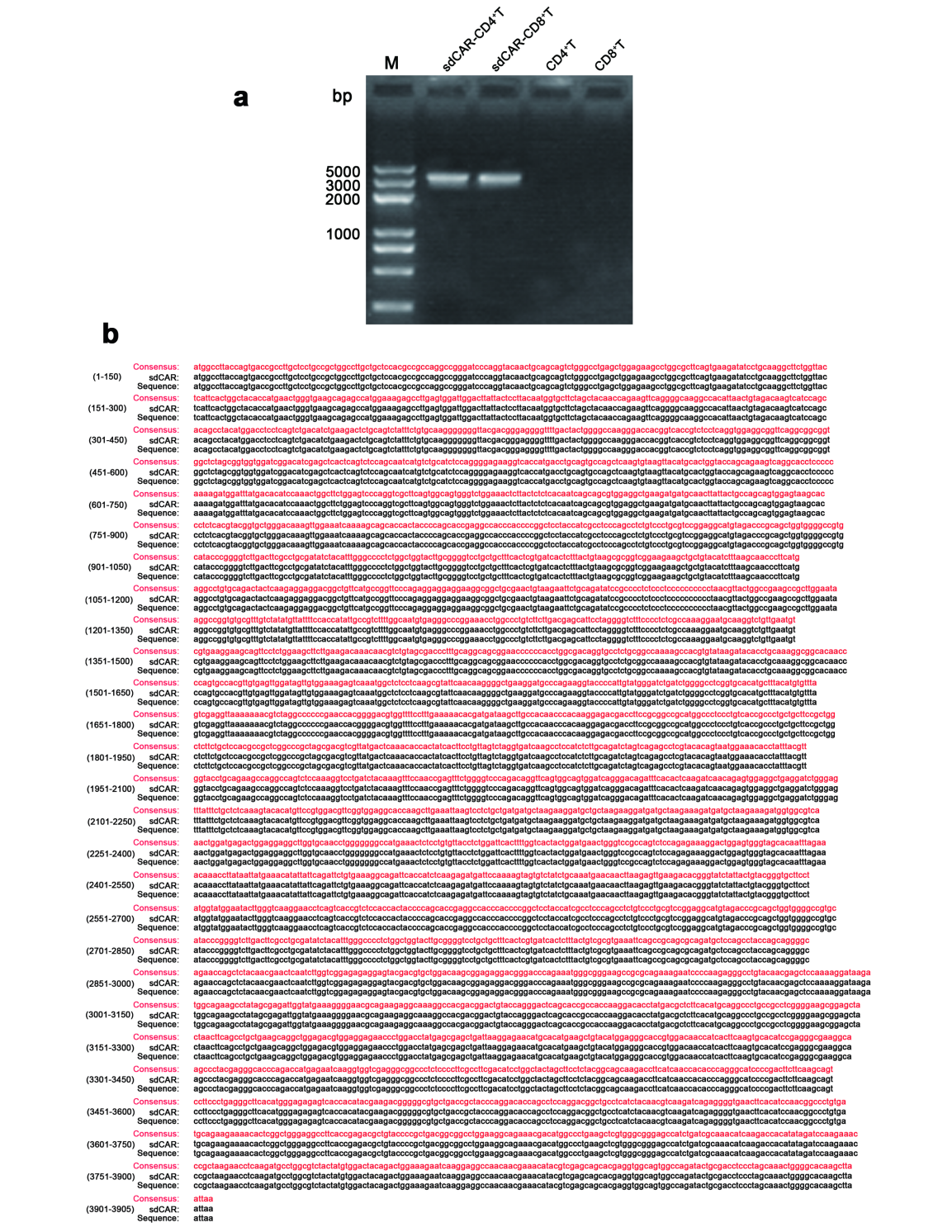
**

**Figure S1.** Identification of sdCAR structure gene in sdCAR-engineered T cells. **a** The cell RNA was extracted, and then reversed into cDNA, the sdCAR fusion gene was amplified by PCR in sdCAR-engineered T cells. **b** The above PCR amplification products were then sequenced and the results showed that the sdCAR fusion gene in both sdCAR-CD4^+^ T cells and sdCAR-CD8^+^ T cells was consistent with the designed genes by sequencing (sdCAR, gene sequencing result; Sequence, designed fusion gene).

**

**

**Figure S2.** Detection of cognate and noncognate antigen expression on K562 cells or HT29 cells. **a** With use of an anti-MSLN antibody and anti-CEA antibody, the expressions of MSLN and CEA on the wild type K562 cells or HT29 cells were determined by flow cytometry. Flow cytometry analysis of cells stained with isotype control antibodies (left panel) or simultaneous staining with an anti-CEA-FITC antibody and an anti-MSLN-PE antibody (right panel). **b** Detection of CEA or MSLN expression on CEA^+^ K562 cell or MSLN^+^ K562 cell, respectively. The positive rate of CEA^+^ K562 cell was determined to be 84.93% (left panel) and the positive rate of MSLN^+^ K562 cell was determined to be 81.34% (right panel). **c** Detection of MSLN expression on MSLN^+^ HT29 cell. The positive rate of MSLN^+^ HT29 cell was determined to be 90.67%.

**
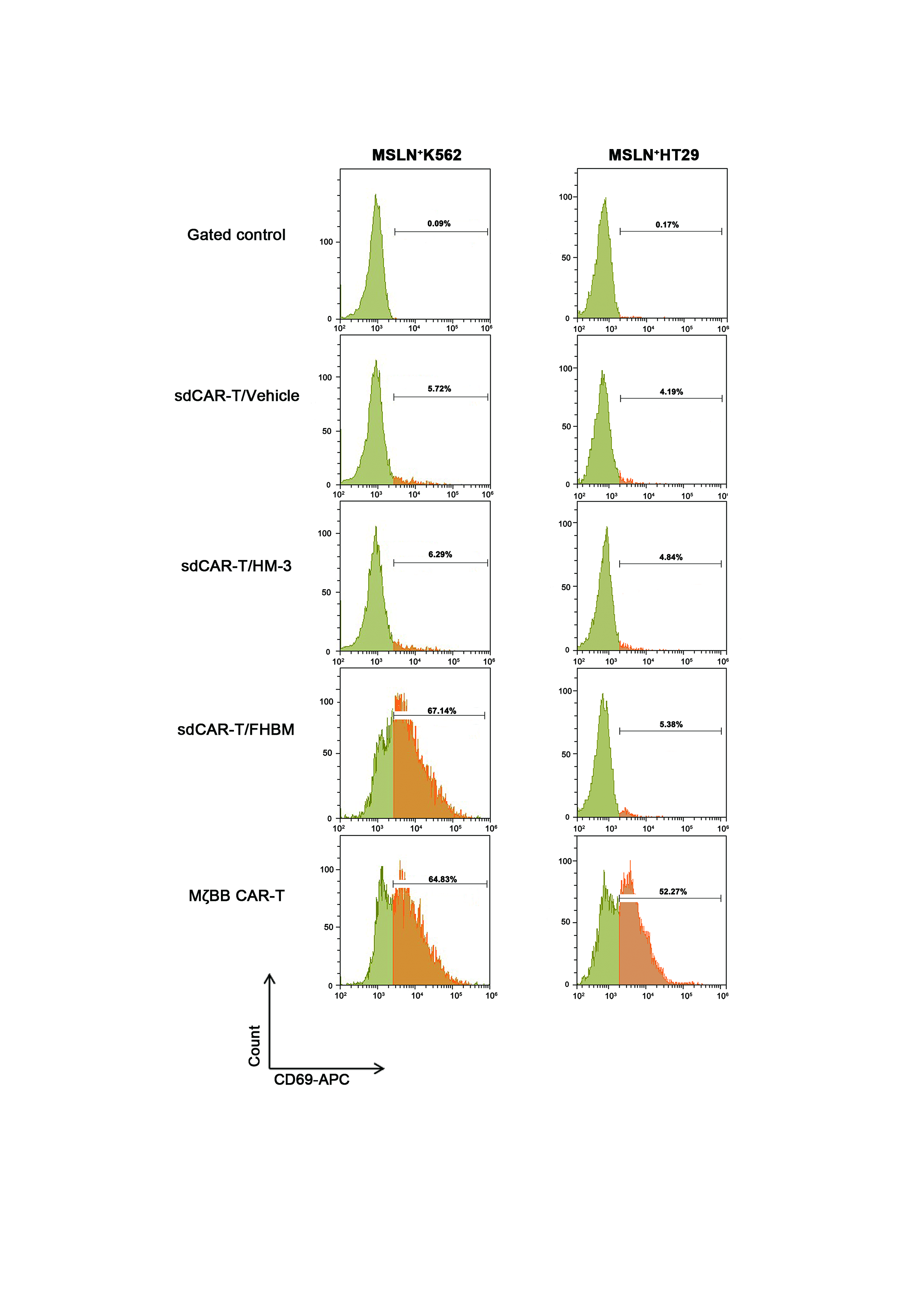
**

**Figure S3.** Expression levels of CD69 on activated CAR-T cells. The expression of CD69 molecule was detected on modified-T cells expressing sdCAR after co-culture with MSLN^+^ K562 cells or MSLN^+^ HT29 cells in presence of PBS, HM-3 or FHBM, respectively. Meanwhile, the level of CD69 expression on MζBB CAR-T cells was detected.


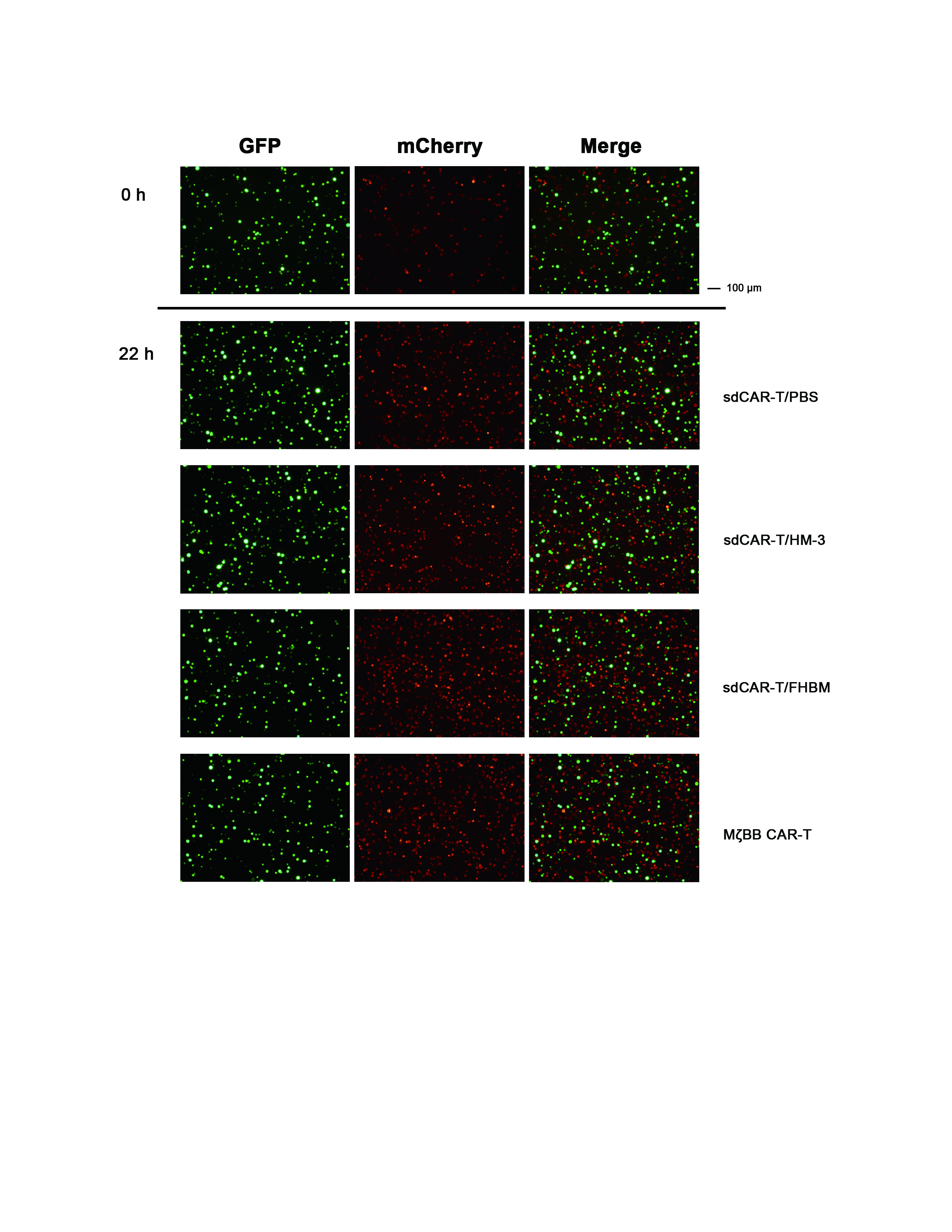


**Figure S4.** Representative fluorescence images of cognate antigen and non-cognate target cells after a 22-h incubation with sdCAR-T cells or MζBB CAR-T cells. With distinct fluorescence protein expression, the mixed target cells were observed by fluorescence microscopy in each sample (n = 3). The topper sample represents the mixture of MSLN^+^ and CEA^+^ K562 cells at the initial state (0 h). The lower four samples represent the target cells incubated with modified-T cells after 22 hours. Left panel: GFP imaging (MSLN^+^ K562 cell); Middle panel: mCherry imaging (CEA^+^ K562 cell); Right panel: merge imaging.


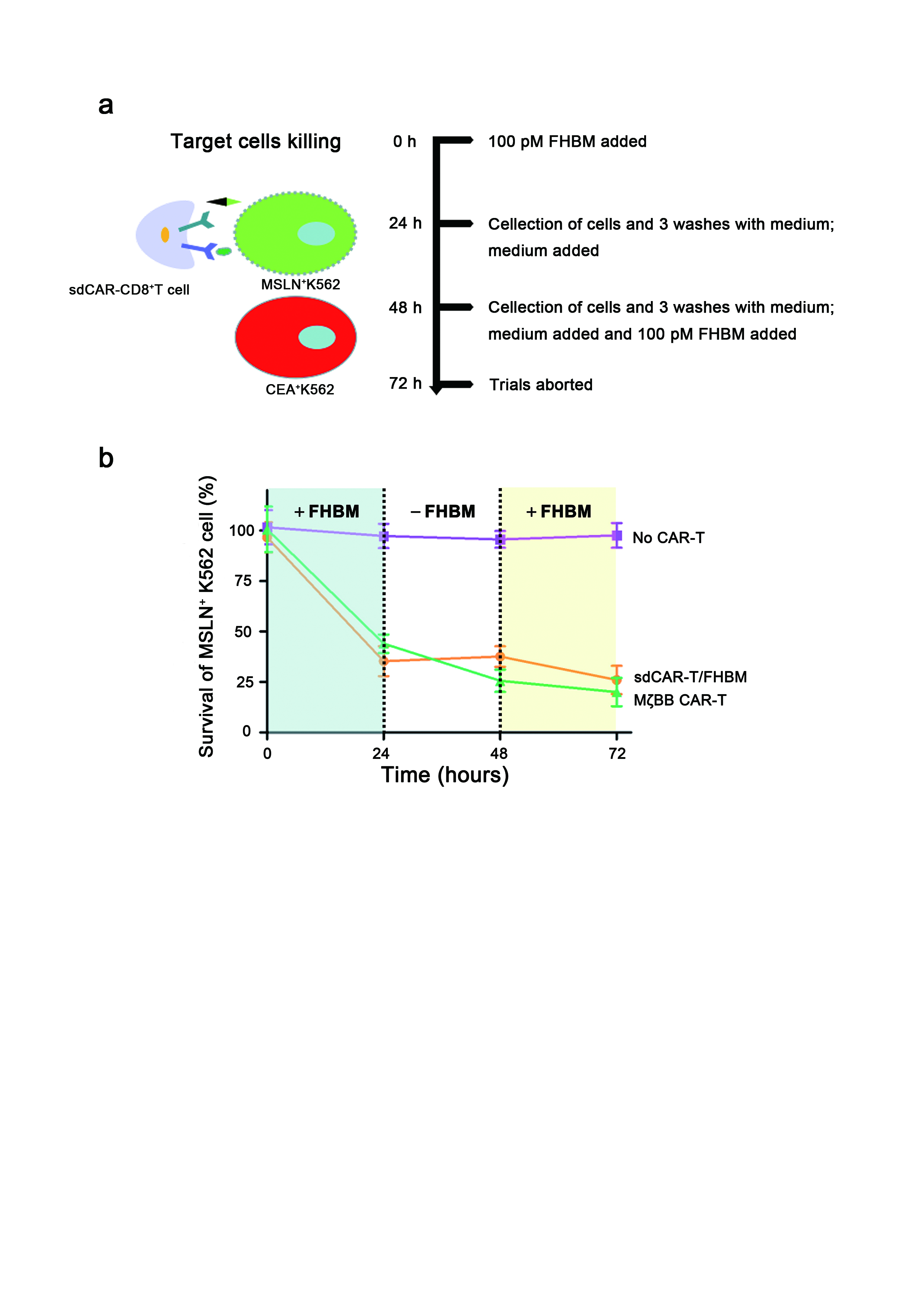


**Figure S5.** sdCAR-T cell function was strictly dependent on FHBM. The experiment consisted of 3 stages to implement the “presence-absence-presence” sequence of exposure to FHBM. A target cell mixture containing CEA^+^ and MSLN^+^ K562 cells at a 1:1 ratio was co-cultured with sdCAR-T cells at a 1:5 ratio in 24-well plates. At designed time points (0 h, 24 h, 48 h and 72 h), the samples were collected for flow cytometry analysis. At the 24 and 48 hour, total cells were washed 3 times with fresh medium and cultured in medium supplemented with IL-2 at 80 IU/mL initially. (n = 3, error bars denote standard deviation.)

**
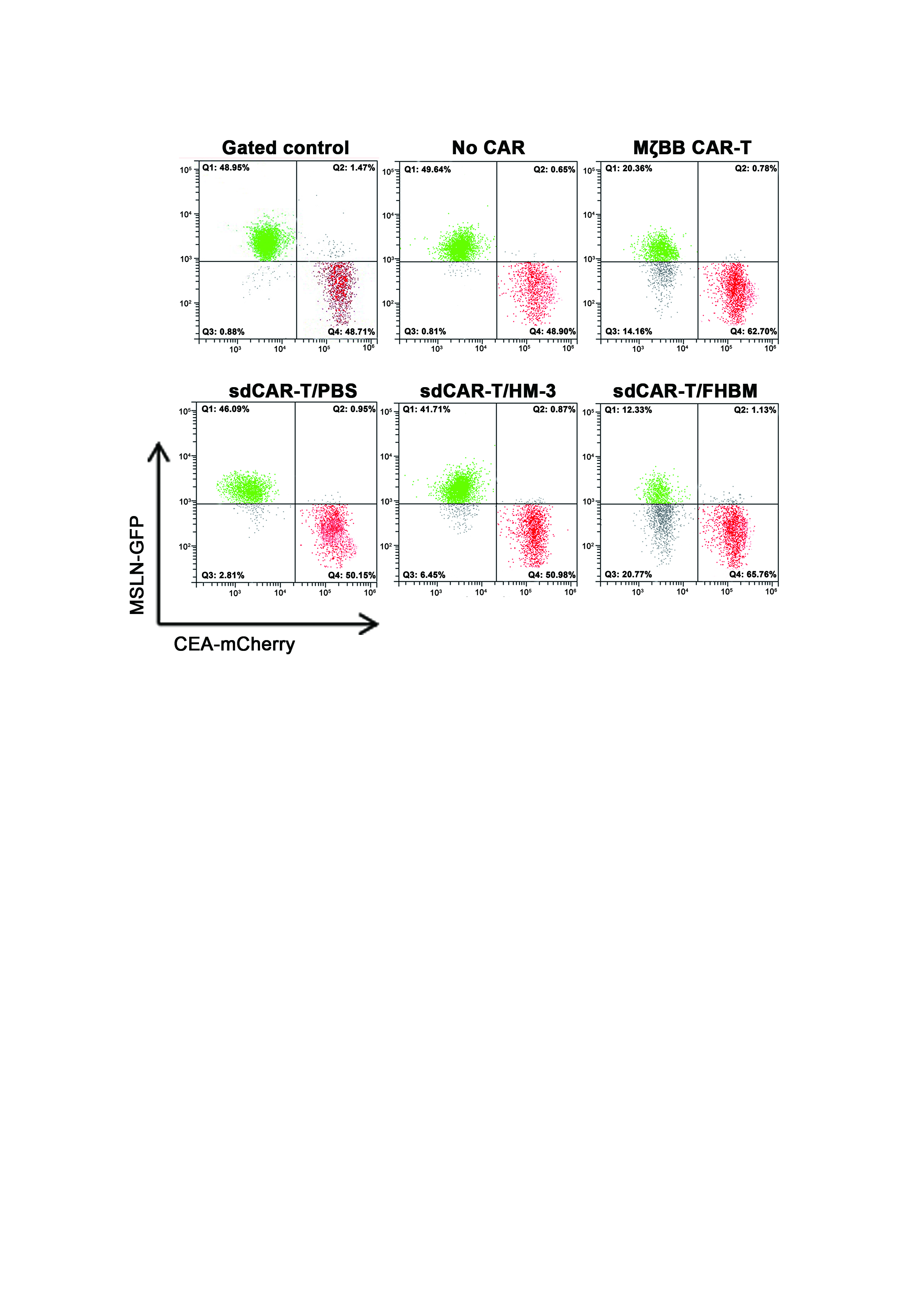
**

**Figure S6.** Schematic representation of flow cytometry-based target cell killing assay *in vivo*. As expression of MSLN and CEA was coupled with the selective expression of GFP and mCherry, the two target cells could be independently identified by flow cytometry. Survival of MSLN^+^ K562 cells was calculated based on the collected total cells by flow cytometry with the methods described in Figure 4B.


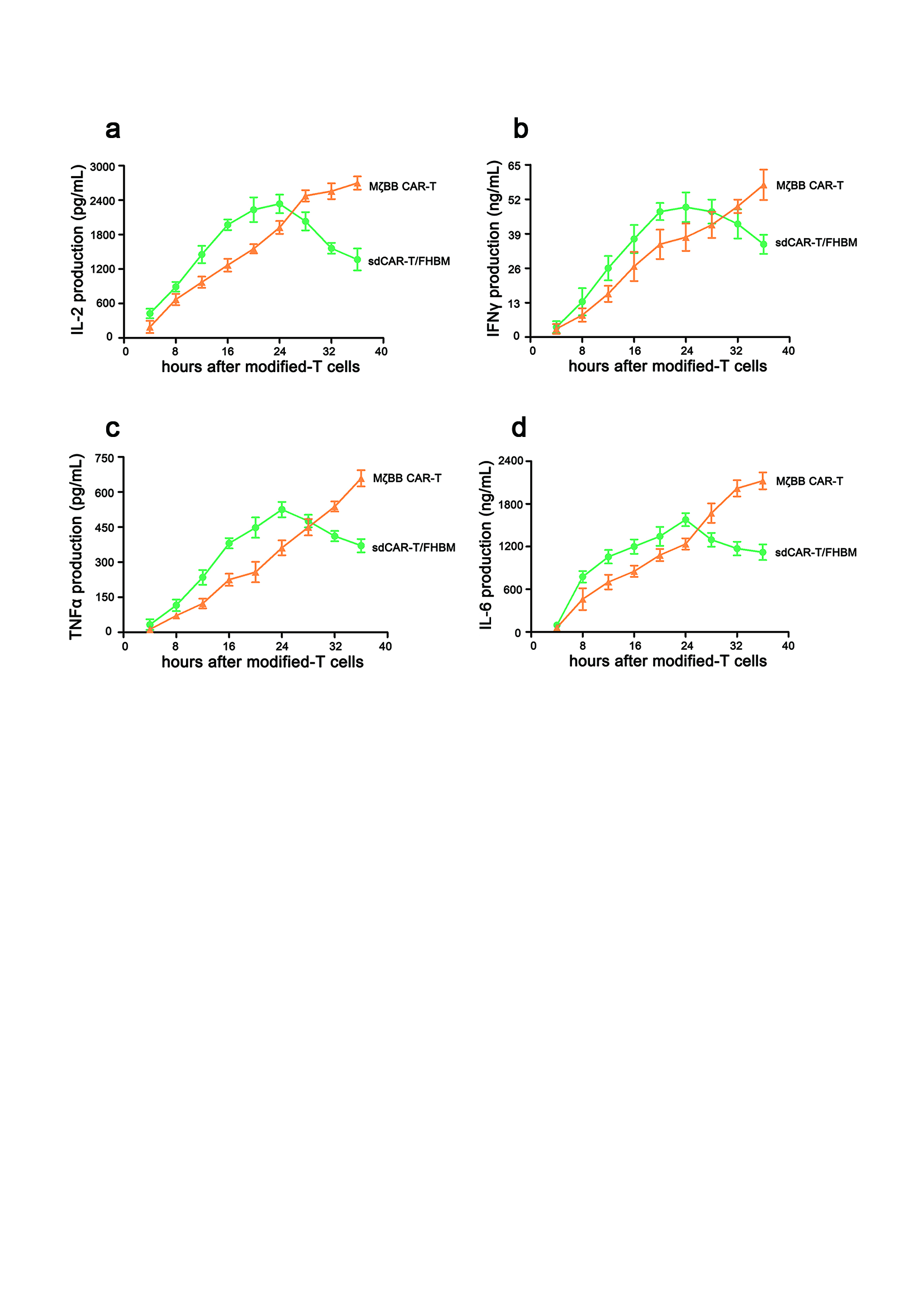


**Figure S7.** The cytokine levels over time in mice after injection of modified-T cells. The levels of cytokines in mice, including IL-2, IFNγ, IL-6, and TNFα, were examined every 4 hours. Compared with treatment of MζBB CAR-T cells, there are some different trends of cytokines in mice with treatment of sdCAR-T cells together with FHBM. (n = 5, error bars denote standard deviation.)

**
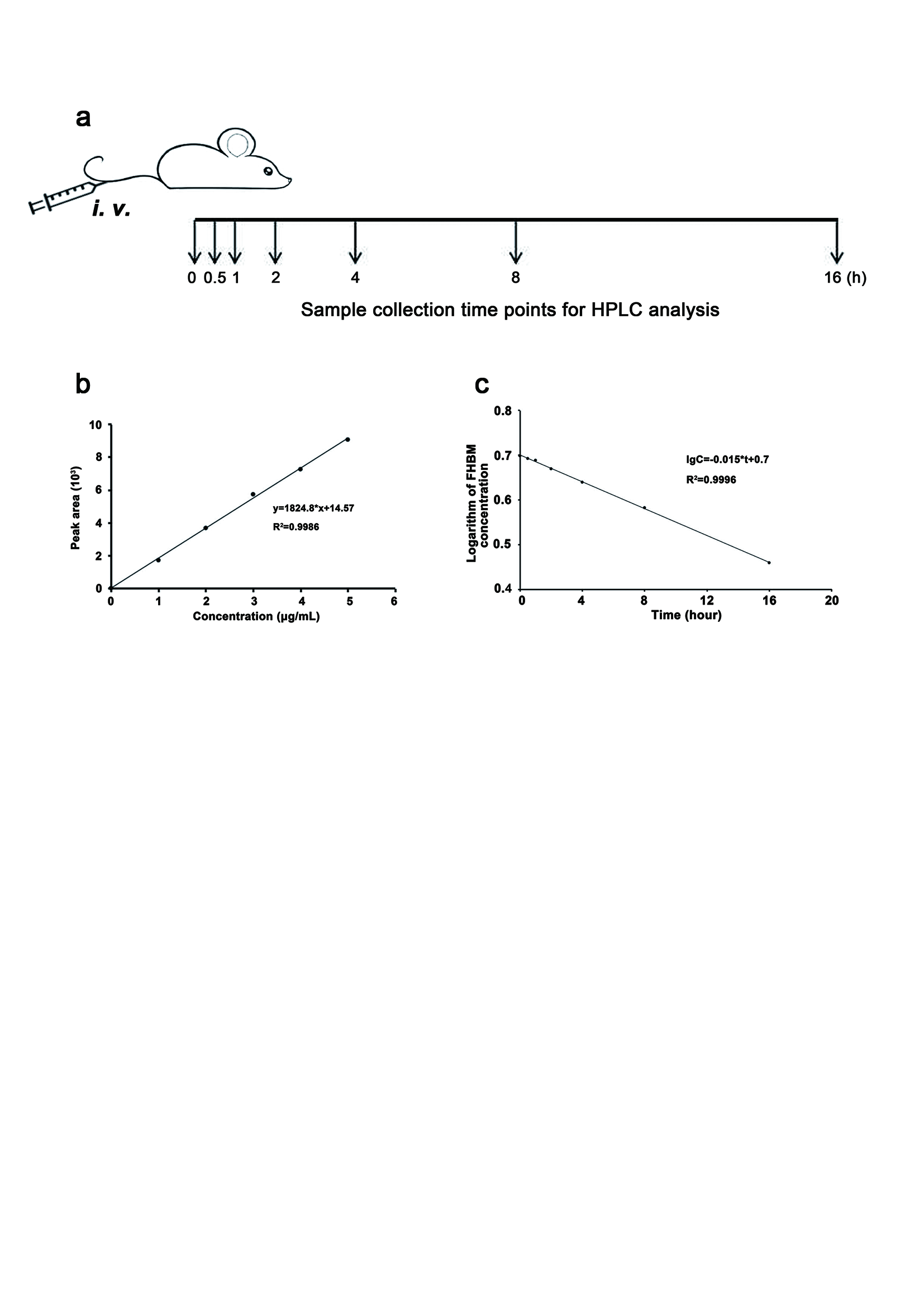
**

**Figure S8.** Calculate the value of the half-life of FHBM. **a** A schematic of the mouse treatment strategy used in the experiment for calculating the half-life of FHBM. The mice with AsPC-1 cells were injected with 20 μg FHBM, and 100 μL of venous blood were collected at the indicated time points. **b** The standard curve of FHBM was emulated as follows: Y = 1824.8*X＋14.57 (with a correlation coefficient of 0.9968), wherein Y indicates peak area and X indicates compound concentration. **c** According to the standard curve, the sample concentrations were calculated at the indicated time points. To confirm that the reaction is first order, we tried to fit the logarithm of concentration versus time. With the least-squares analysis, the regression equation was obtained as follows: lgC = 0.7－0.015*T (with a correlation coefficient of 0.9996), wherein C indicates compound concentration and T indicates time.


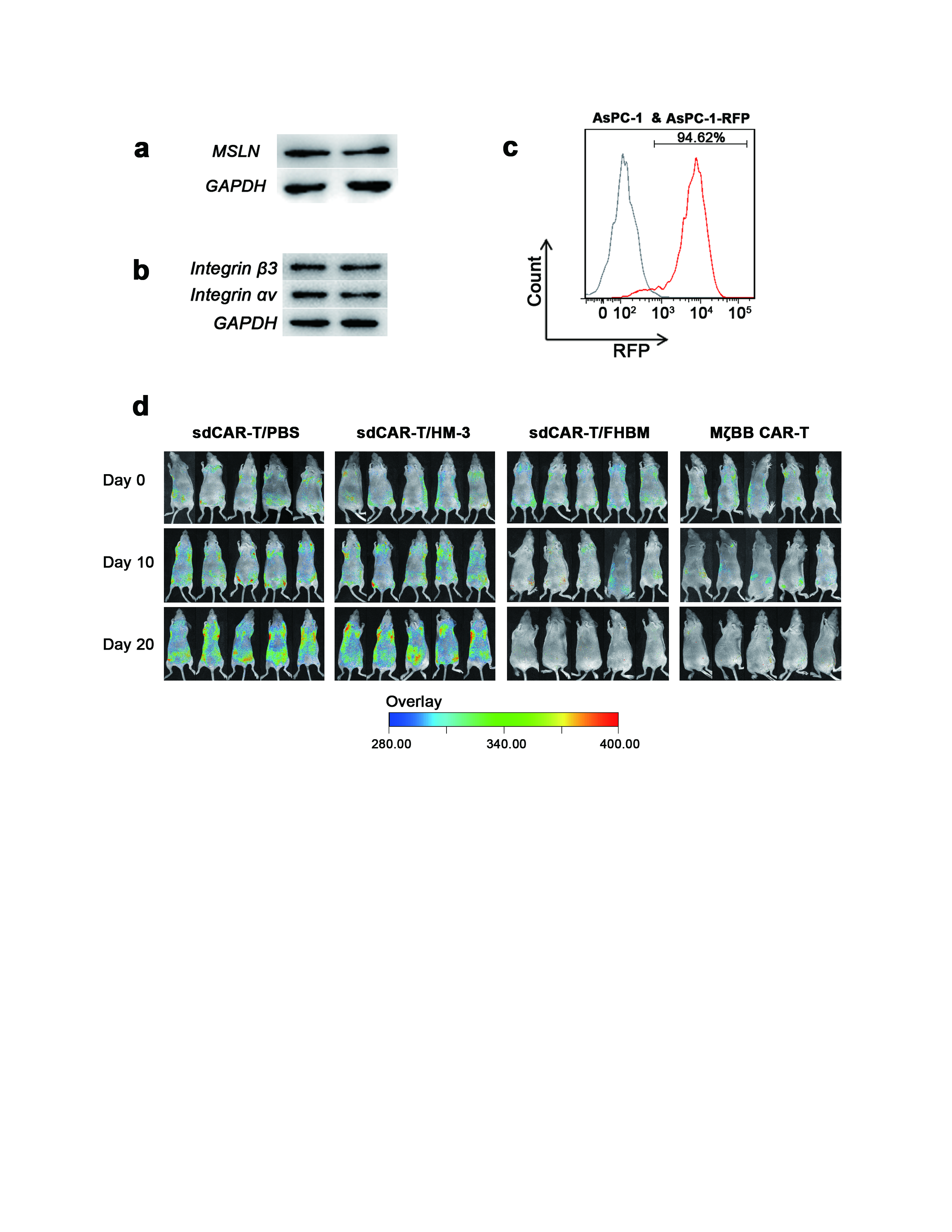


**Figure S9.** sdCAR-T cell cytotoxicity for solid tumor in xenograft. **a** Western blot results confirmed the expression of cognate antigen (MSLN) on AsPC-1 cells. **b** Western blot results showed that the integrin αvβ3 was highly expressed on the surface of AsPC-1 cells. **c** We evaluated the transduction efficiency by flow cytometry with endogenous RFP expression in engineered AsPC-1 cells (94.62%). **d** The results showed that sdCAR-T cells combined with a bifunctional molecule have significant cytotoxicity for AsPC-1 cells expressing integrin αvβ3 and MSLN in xenograft.
